# Biomarkers of Cerebral Injury and Inflammation in Pediatric Tuberculous Meningitis

**DOI:** 10.1093/cid/cix540

**Published:** 2017-06-09

**Authors:** Ursula K Rohlwink, Katya Mauff, Katalin A Wilkinson, Nico Enslin, Emmanuel Wegoye, Robert J Wilkinson, Anthony A Figaji

**Affiliations:** 1 Division of Neurosurgery,; 2 Wellcome Centre for Infectious Diseases Research in Africa, Institute of Infectious Diseases and Molecular Medicine, Department of Medicine, and; 3 Department of Statistical Science, University of Cape Town, South Africa; and; 4 Francis Crick Institute and; 5 Department of Medicine, Imperial College London, United Kingdom

**Keywords:** pediatric tuberculous meningitis, biomarkers, S100B, NSE, GFAP

## Abstract

**Background:**

Tuberculous meningitis (TBM) leads to death or disability in half the affected individuals. Tools to assess severity and predict outcome are lacking. Neurospecific biomarkers could serve as markers of the severity and evolution of brain injury, but have not been widely explored in TBM. We examined biomarkers of neurological injury (neuromarkers) and inflammation in pediatric TBM and their association with outcome.

**Methods:**

Blood and cerebrospinal fluid (CSF) of children with TBM and hydrocephalus taken on admission and over 3 weeks were analyzed for the neuromarkers S100B, neuron-specific enolase (NSE), and glial fibrillary acidic protein (GFAP), in addition to multiple inflammatory markers. Results were compared with 2 control groups: patients with (1) a fatty filum (abnormal filum terminale of the spinal cord); and (2) pulmonary tuberculosis (PTB). Imaging was conducted on admission and at 3 weeks. Outcome was assessed at 6 months.

**Results:**

Data were collected from 44 patients with TBM (cases; median age, 3.3 [min–max 0.3–13.1] years), 11 fatty filum controls (median age, 2.8 [min–max 0.8–8] years) and 9 PTB controls (median age, 3.7 [min–max 1.3–11.8] years). Seven cases (16%) died and 16 (36%) had disabilities. Neuromarkers and inflammatory markers were elevated in CSF on admission and for up to 3 weeks, but not in serum. Initial and highest concentrations in week 1 of S100B and NSE were associated with poor outcome, as were highest concentration overall and an increasing profile over time in S100B, NSE, and GFAP. Combined neuromarker concentrations increased over time in patients who died, whereas inflammatory markers decreased. Cerebral infarcts were associated with highest overall neuromarker concentrations and an increasing profile over time. Tuberculomas were associated with elevated interleukin (IL) 12p40, interferon-inducible protein 10, and monocyte chemoattractant protein 1 concentrations, whereas infarcts were associated with elevated tumor necrosis factor α, macrophage inflammatory protein 1α, IL-6, and IL-8.

**Conclusions:**

CSF neuromarkers are promising biomarkers of injury severity and are predictive of mortality. An increasing trend suggested ongoing brain injury, even though markers of inflammation declined with treatment. These findings could offer novel insight into the pathophysiology of TBM.

## Summary

Neurospecific biomarkers were elevated in pediatric tuberculous meningitis and showed an increasing temporal profile in patients who died, whereas markers of inflammation decreased in all patients regardless of outcome. Secondary injury mechanisms initiated by inflammation likely cause ongoing brain damage.

Tuberculous meningitis (TBM) is a severe form of cerebral tuberculosis leading to high rates of death and disability in children [1–3]. The resulting cerebral tissue injury is an important determinant of poor outcome and is considered a consequence of the host immune response. The prolific inflammatory process causes extensive basal subarachnoid exudate and vascular involvement, with external compression, vasospasm, and endovasculitis [4]. Additionally, it often obstructs the flow of cerebrospinal fluid (CSF), precipitating hydrocephalus. The consequent raised intracranial pressure (ICP) aggravates the already decreased cerebral blood flow and increases the risk of brain ischemia and infarction.

Quantification of the degree of cerebral injury is inexact: Disease severity is commonly evaluated by clinical and radiologic findings. However, these have limited utility; several reversible and irreversible factors influence clinical status and imaging findings usually manifest once the damage is already permanent. Advanced tools to assess cerebral injury severity and prognosis are lacking. Inflammatory markers have been studied in TBM [5–12]; however, the extent of inflammation as detected by these markers may not correlate with the extent of cerebral tissue injury. Direct markers of cerebral tissue injury are elevated in TBM [13, 14] but have not been examined in detail or in association with markers of inflammation. Reliable markers of neurological tissue injury could serve as biomarkers to assess injury severity, monitor disease resolution or deterioration, direct treatment, prognosticate, and as surrogate end points to help develop novel treatments. Literature on neurotissue markers in various central nervous system (CNS) pathologies (including infection, trauma, stroke, hemorrhage, and degenerative disease) has identified significant relationships between brain injury and/or poor clinical outcome and elevated concentrations of neuron-specific enolase (NSE) (a marker of neuronal injury), S100B, and glial fibrillary acidic protein (GFAP) (both markers of astroglial injury) [15]. Given their promise in other forms of brain injury, we examined the concentrations and temporal profiles of these 3 markers of neurological tissue injury in serum and in lumbar and ventricular CSF compared with controls. These were combined with markers of inflammation, and examined in association with clinical and radiologic outcome in children with TBM.

## METHODS

This study was approved by the Institutional Review Board of the University of Cape Town (HREC 318/2010). Informed consent was obtained from the parents/guardians of all participants.

### Patient Cohort

Over a 3-year period (October 2010–August 2013), study participants from the Red Cross War Memorial Children’s Hospital, Cape Town, were enrolled in this prospective study. TBM cases comprised children admitted for definite or probable TBM according to a published case definition [16]. Cases were limited to patients with associated hydrocephalus as they undergo repeated CSF diversion procedures as part of treatment. All cases were treated with the standard antimicrobial regimen and adjunctive prednisone [17]. Hydrocephalus was treated according to a published protocol [18]. Demographic and clinical data were collected as previously described [19]. Clinical outcomes according to the Pediatric Cerebral Performance Category Scale (PCPS) [20] (poor outcome [PCSP 4–6: severe disability or death]; good outcome [PCPS 1–3: normal to mild/moderate disability]) and mortality at 6 months were recorded. Radiological data were collected from admission and follow-up computed tomography (CT) brain scans, 3-week magnetic resonance imaging (MRI) of the brain and spine, and magnetic resonance angiography of the cerebral vessels [21]. Control patients included (1) children admitted for elective surgery for a fatty filum terminale of the spinal cord (a congenital abnormality not associated with neurological injury or inflammation), and (2) children clinically diagnosed with and treated for pulmonary TB (PTB).

### Sample Collection

Samples from cases included lumbar CSF, ventricular CSF (from the lateral ventricles), and serum collected concurrently with clinically indicated procedures on admission and once weekly for 3–4 weeks (Supplementary Data 1). For the fatty filum controls, blood was drawn prior to surgery and lumbar CSF was taken before the dura was opened. Blood from PTB controls was taken before treatment initiation or within 48 hours thereof. Samples were stored at –80°C for batch analysis. Commercially available enzyme-linked immunoassays were used to analyze neuromarkers S100B (Merck Millipore, Billerica, Massachusetts), NSE (DRG Diagnostics, Marburg, Germany) and GFAP (Merck Millipore) according to manufacturer instructions. Interleukin (IL) 1β, IL-1 receptor antagonist (RA), IL-6, IL-10, IL-12p40, tumor necrosis factor alpha (TNF-α), interferon gamma (IFN-γ), IL-8, growth-regulated oncogene (GRO), monocyte chemoattractant protein 1 (MCP-1), interferon-inducible protein 10 (IP-10), macrophage inflammatory protein 1α (MIP-1α), vascular endothelial growth factor (VEGF), and regulated upon activation, normal T-cell–expressed and secreted protein (RANTES) were analyzed on the Bio-Plex platform (Bio-Rad Laboratories, Hercules, California), using customized Milliplex kits (Millipore, St Charles, Missouri), according to manufacturer instructions. Samples were excluded when they were taken within 48 hours after neurosurgical procedures, were not immediately refrigerated or processed within 12 hours of collection, or exhibited hemolysis or xanthochromia (NSE only) (Supplementary Data 1).

### Imaging Data

As shown in the Supplementary Data 3, hydrocephalus was defined as mild (visible temporal horns, rounding of the third ventricle [V3]), moderate (all ventricles dilated, or at least triventricular in the case of aqueduct obstruction, no transependymal fluid shift), or severe (markedly dilated ventricles, fluid shift, and loss of sulcal markings). Infarcts were recorded as unilateral or bilateral, and their size was graded as single (lacunar), multiple/large (multiple or large branch infarcts), and large territory (encompassing middle cerebral artery (MCA), anterior cerebral artery (ACA), posterior cerebral artery (PCA) territories). Tuberculomas were classified by number (single or multiple when >1). Scans were evaluated by 3 assessors blinded to patient outcome.

### Data Analysis

Descriptive statistics were compiled for samples at each time point. Neuromarker concentrations of TBM cases were compared to controls using Mann-Whitney *U* test with a Holm *P* value adjustment. The association between initial (admission or week 1 when no admission data were available) neuromarker concentrations and patient characteristics (age, sex, admission symptoms) was evaluated using the χ^2^ or Fisher exact test. The correlation between neuro- and inflammatory markers and CSF glucose, chloride, protein, and white cell count was examined using Spearman correlation. Using the Kruskal-Wallis test, findings on admission CT scan (Supplementary Data 3) were analyzed in association with initial (admission) CSF neuro- and inflammatory marker concentrations. Moreover, radiologic features overall (admission and follow-up CT/MRI) were examined in association with highest overall concentrations of the neuro- and inflammatory markers, and the change in neuromarker concentrations between admission and week 2 (ΔS100B, ΔNSE, ΔGFAP) as an indicator of early change.

Outcome analysis focused on neuromarkers:

- Univariate analysis: The relationship between neuromarker concentrations and outcome was examined using Mann-Whitney *U* test. Various neuromarker indices including initial, highest in week 1, highest overall throughout hospitalization, and Δ were examined to control for the limitations of each individual index.
- PCA: *z* scores combining all 3 neuromarkers (Z-Neuro) or all 14 inflammatory markers (Z-Inflammatory) for each patient were derived as a statistical approximation of a single index for neurological injury or inflammation; time was added to this analysis to generate 3-dimensional PCA biplots, and mortality was indicated on the plot (Supplementary Data 2).
- Receiver operating characteristic (ROC) analysis: ROC curves were constructed to identify the thresholds of highest S100B, NSE, and GFAP as well as ΔS100B, ΔNSE, and ΔGFAP, which best predicted outcome.
- Multivariate analysis: Logistic regression models for poor outcome were constructed for ΔZ-Neuro, ΔS100B, ΔNSE, and ΔGFAP as these indices demonstrated the strongest outcome association on univariate analysis. Covariates were drawn from admission and radiologic characteristics, which were significantly associated with clinical outcome [19].

## RESULTS

Forty-four TBM cases (median age, 3.3 [min–max 0.3–13.1] years) were enrolled (Table 1). The culture positivity yield was 56.4%, and no drug resistance was reported. Hydrocephalus was noncommunicating in 7% of patients. Thirty-two patients (72.7%) had a good clinical outcome, and 7 patients died (16%). Infarcts were reported in 66% of patients, tuberculomas in 11%, and spinal pathology in 76% (Table 2). Eleven fatty filum controls (median age, 2.75 [min–max 0.75–8] years) and 9 PTB controls (median age, 3.67 [min–max 1.25–11.83] years) were enrolled and were similar to cases in age and sex distribution. PTB controls were treated according to the standard medical regimen.

**Table 1.** Demographic and Clinical Characteristics

Characteristic	Value
TBM cases	
Demographic characteristics	
** **Age, y, median (min–max)	3.3 (0.3–13.1)
** **0–2	19 (43.2)
** **3–5	18 (40.9)
** **>5	7 (15.9)
** **Sex (male)	28 (63.6)
** **Admission characteristics	
** **MRC TBM staging on admission	
** **1	4 (9)
** **2	31 (70.5)
** **3	9 (20.5)
** **Symptom duration, d, median (min–max)	7.5 (1–90)
** **Vomiting	24 (54.5)
** **Headache (n = 37)^a^	15 (40.1)
** **Fever	30 (68.2)
** **Seizures	14 (31.2)
** **Focal neurology^b^	21 (47.7)
** **Altered level of consciousness	40 (90.1)
** **Meningism	34 (77.2)
** **Bulging fontanelle (n = 9)^c^	5 (55.6)
** **HIV infection (n = 43)^d^	2 (4.7)
** **Diagnostics	
** **CSF TB culture or AFB positive (n = 39)^e^	22 (56.4)
** **Outcome	
** **Good clinical outcome^f^	32 (72.7)
** **Poor clinical outcome^g^	12 (27.2)
** **Mortality^h^	7 (16)
** **Controls	
** **Age, y , median (min–max)	
** **Fatty filum controls	2.75 (0.8–8)
** **Pulmonary TB controls	3.67 (1.3–11.8)
** **Sex (male)	
** **Fatty filum controls	8 (72.7)
** **Pulmonary TB controls	5 (55.6)

Data are presented as No. unless otherwise indicated.

Abbreviations: AFB, acid-fast bacilli; CSF, cerebrospinal fluid; HIV, human immunodeficiency virus; MRC, British Medical Research Council; TB, tuberculosis; TBM, tuberculous meningitis.

^a^Preverbal children <1.5 years of age excluded.

^b^Focal neurology included aphasia, absence of pupillary response, paresis, cranial nerve palsies, and paresis.

^c^For children with open fontanelles.

^d^Only 43 patients had HIV testing performed.

^e^Only 39 patients had CSF sent for TB diagnostics, in 5 patients TB culture was not requested in error or the volume of CSF was insufficient.

^f^Pediatric Cerebral Performance Category Scale (PCPS) 1–3: normal to mild/moderate disability.

^g^PCPS 4–6: severe disability and death.

^h^Four of these patients died within 10 days of hospital admission.

**Table 2.** Summary of Radiologic Features Overall

Radiology Features	Admission CT (n = 44)	Follow-up CT/MRI (n = 43)
Hydrocephalus present	44 (100)	24 (61.5)^a^
Severity		
Mild	11 (25)	18 (75)
Moderate	24 (54.5)	6 (25)
Severe	9 (20.5)	2 (8.3)
Basal enhancement	41 (95.5)	38 (100)^b^
Infarcts present	9 (20.5)	29 (67.4)
Unilateral	6 (66.7)	14 (48.3)
Bilateral	3 (33.3)	15 (51.2)
Infarct size		
Small	3 (33.3)	10 (34.5)
Moderate	6 (66.7)	16 (55.2)
Large	0	3 (10.3)
MRA pathology detected	…	16 (55.2)^c^
Tuberculoma present	3 (6.8)	23 (59)^d^
Single	1 (33.3)	10 (43.5)
Multiple	2 (66.7)	13 (56.5)
Spinal pathology present		
Spinal arachnoiditis and/or exudate	…	24 (75.8)
Spinal tuberculoma	…	6 (18.2)

Data are presented as No. (%). Frequency of radiologic features reported on admission CT scans and at follow-up: 43 patients had follow-up imaging during their hospital stay. Follow-up CT/MRI includes CT scans of 4 patients who died within 10 days of admission and MRI of 39 patients at 26 ± 11 days postadmission; 1 patient had a follow-up MRI at 3 months.

Abbreviations: CT, computed tomography; MRA, magnetic resonance angiography; MRI, magnetic resonance imaging.

^a^Hydrocephalus was treated with external ventricular drains or ventriculoperitoneal shunts in all 4 patients who died early.

^b^Thirty-eight of the 39 patients with follow-up MRI received contrast.

^c^MRA was performed in 29 patients at 3 weeks as this imaging was a late addition to the study protocol.

^d^Follow-up head CT scans of the 4 deceased patients were not contrast-enhanced; tuberculomas were only reported on follow-up MRI in the remaining 39 patients.

**Figure 1.**
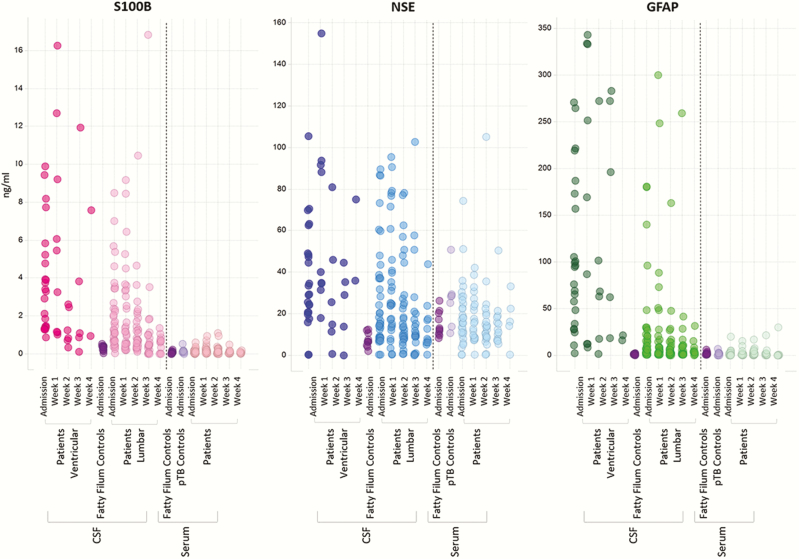
Neuromarker concentrations for cases over time. Concentrations (ng/ml) for neuromarkers S100B, NSE and GFAP in ventricular CSF, lumbar CSF and serum are plotted from admission to week 4 for all TBM cases. Ventricular samples from week 4 represent only 2 patients – the elevated concentrations belonged to a patient who had failed medical treatment for their hydrocephalus and required a ventriculo-peritoneal shunt. Concentrations for fatty filum and pulmonary TB (pTB) controls are also shown.

Descriptive statistics for neuromarkers for each time point are shown in Figure 1 and included in Supplementary Data 4 and 5 (inflammatory data also presented). CSF neuro- and inflammatory marker concentrations were greater in cases than controls, but serum values were not (Supplementary Data 4). CSF neuro- and inflammatory marker concentrations in the CSF remained greater than controls for up to 4 weeks. Overall neuromarker concentrations were greater in ventricular than lumbar CSF, whereas inflammatory marker concentrations were greater in lumbar than ventricular CSF (Figure 1 and Supplementary Data 5 and 6).

Neuromarker concentrations were not significantly associated with patient demographic or admission characteristics. Only lumbar GFAP was significantly more elevated in patients who were culture positive (*P* < .05). Neuro- and inflammatory markers were not significantly associated with CSF glucose, chloride, protein, or white cell count.

### Association With Radiology

#### Admission CT Scan

**Figure 2.**
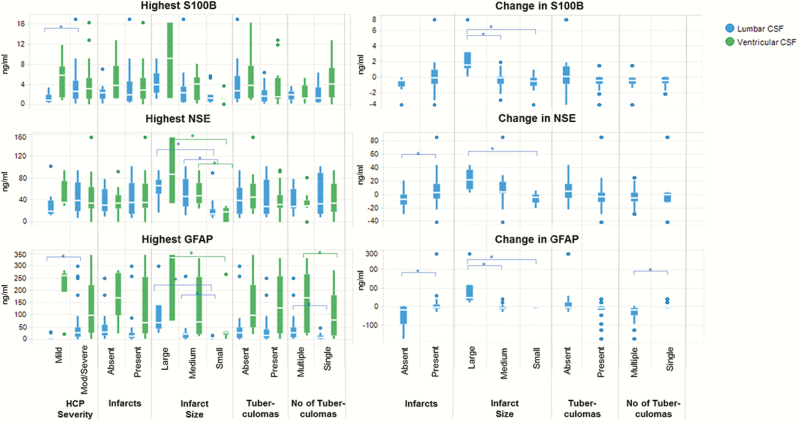
Association between radiology overall and neuromarker concentrations. These box and whisker plots demonstrate the median, interquartile range, and range of the highest neuromarker concentrations (panel 1: lumbar and ventricular cerebrospinal fluid [CSF]) and the change between admission and week 2 (panel 2: lumbar CSF only; a positive value indicates an increase over time) for various radiologic features recorded across admission and in-hospital follow-up scans (overall computed tomography and magnetic resonance imaging). Statistically significant results are indicated by a square bracket and *. Hydrocephalus was scored on admission scans and dichotomized as mild and moderate/severe. There were very few patients with mild hydrocephalus. Abbreviations: CSF, cerebrospinal fluid; GFAP, glial fibrillary acidic protein; HCP, hydrocephalus; NSE, neuron-specific enolase.

The highest lumbar GFAP and S100B concentrations were greater in moderate-to-severe than in mild hydrocephalus (*P* < .01 and *P=* .02, respectively; Figure 2). Ventricular TNF-α and IFN-γ concentrations were higher in patients with mild enhancement (*P=* .05 and *P* = .04, respectively) or no infarcts on their admission scans (*P* = .03 and *P* = .01, respectively).

#### Radiology Overall

The highest lumbar GFAP and NSE were associated with multiple (*P* = .02 and *P <* .01, respectively) and large infarcts compared to small ones (*P <* .01 and *P* = .05, respectively; Figure 2). Highest ventricular GFAP and NSE were also positively associated with larger infarcts (*P* = .04 and *P* = .02, respectively). Lumbar GFAP and NSE concentrations increased from admission to week 2 in patients with infarcts (*P* = .01 and *P* = .03, respectively), particularly with large infarcts relative to small (*P* = .01 and *P* = .01, respectively). Similarly S100B concentrations increased in patients with larger infarcts (*P* = .01). Highest lumbar and ventricular GFAP were associated with multiple tuberculomas (*P* = .04 and *P* = .02, respectively). No association was demonstrated between neuromarkers and spinal disease.

**Figure 3.**
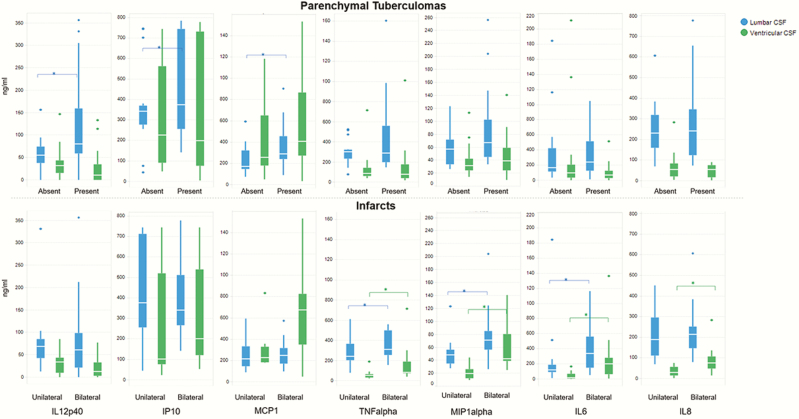
Association between overall radiology and inflammatory marker concentrations. These box and whisker plots demonstrate the median, interquartile range, and range of the highest inflammatory marker concentrations in lumbar and ventricular cerebrospinal fluid (CSF) for various radiologic features recorded across admission and in-hospital follow-up scans (overall computed tomography and magnetic resonance imaging). Statistically significant results are indicated by a square bracket and *. Each row represents the data for individual cytokines (interleukin [IL] 12p40, interferon-inducible protein 10 [IP-10], monocyte chemoattractant protein 1 [MCP-1], tumor necrosis factor alpha [TNF-α], macrophage inflammatory protein 1-alpha [MIP-1α], IL-6, and IL-8) in the presence of parenchymal tuberculomas (top panel) or infarcts (bottom panel).

Parenchymal tuberculomas were positively associated with highest lumbar CSF IL-12p40 (*P* = .04), IP-10 (*P <* .01), and MCP-1 (*P* = .02). Bilateral infarcts were positively associated with highest lumbar and ventricular CSF TNF-α (*P* = .05 and *P* = .01, respectively), MIP-1α (*P* = .03 and *P <* .01, respectively), and IL-6 (*P* = .05 and *P* = .01, respectively) and IL-8 (ventricular CSF only, *P* = .02) concentrations (Figure 3) (multiple testing was not controlled for).

### Association With Outcome

#### Univariate Analysis

**Figure 4.**
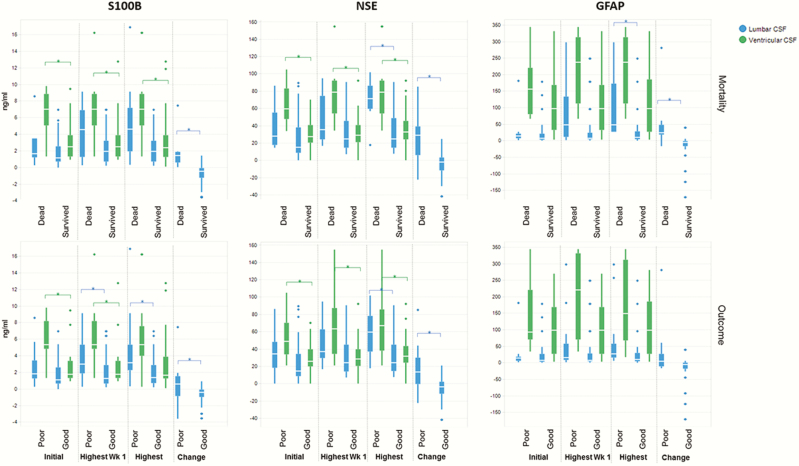
Univariate analysis between outcome and neuromarker indices. These box and whisker plots demonstrate the median, interquartile range, and range of various neuromarker indices (initial/admission concentration, highest concentration in week 1, highest concentration overall during hospitalization and the change between week 2 and admission) in association with mortality (top panel) and dichotomized clinical outcome (bottom panel, pediatric cerebral performance category scale [PCPS] 1–3: normal to mild/moderate disability = good outcome; PCSP 4–6: severe disability or death = poor outcome). Statistically significant results are indicated by a square bracket and *. Abbreviations: CSF, cerebrospinal fluid; GFAP, glial fibrillary acidic protein; NSE, neuron-specific enolase.

The highest lumbar S100B concentrations recorded in week 1 and overall were associated with outcome at 6 months (*P* = .04 and *P* = .03, respectively), and an increasing trend was significantly predictive of mortality and 6-month outcome (*P <* .001 and *P* = .02, respectively) (Figure 4). Initial and highest in week 1 ventricular S100B concentrations were associated with mortality (*P* = .02 and *P* = .03, respectively) and 6-month outcome (*P* = .01 and *P* = .01), and highest overall with mortality (*P* = .02). Highest lumbar NSE overall and an increasing trend were predictive of mortality (*P* = .01 and *P* = .01, respectively) and 6-month outcome (*P* = .01 and *P* = .02, respectively). All ventricular NSE indices were strong predictors of mortality and 6-month outcome (*P ≤* .01). Highest lumbar GFAP overall and an increasing trend were associated with mortality (*P* = .01 and *P* = .003, respectively). Ventricular GFAP was not a predictor of mortality or outcome.

#### PCA Analysis

**Figure 5.**
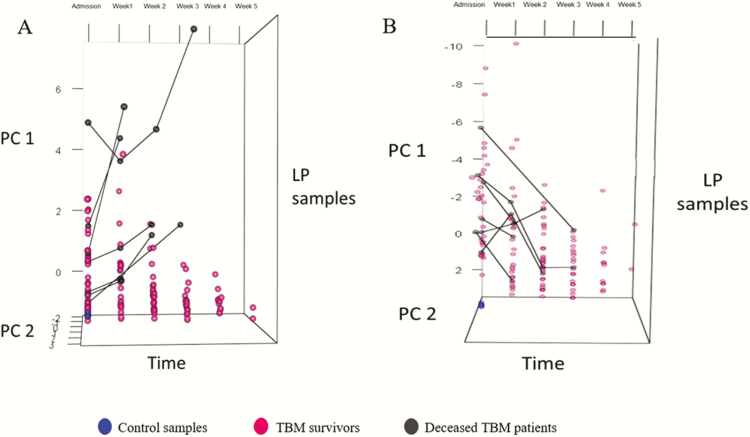
PCA plot: Temporal profile of combined neuromarkers in lumbar puncture (LP) cerebrospinal fluid (CSF) samples. These 3-dimensional principal component analysis (PCA) plots depict the *z* scores for combined neuromarkers (*A*) and combined inflammatory markers (*B*) in lumbar CSF over the duration of sampling (admission to week 5). Each circle represents a patient’s *z* score at that time point. The axes represent principal component 1 (PC1), principal component 2 (PC2), and time. *A*, Patients who died (indicated by circles joined by lines) had increasing neuromarker trends, whereas survivors demonstrated a decline in concentrations over time. *B*, Overall, patients who died (indicated by circles joined by lines) had decreasing inflammatory marker trends, similar to survivors. Abbreviation: TBM, tuberculous meningitis.

PCA plots of the temporal profile of combined neuromarkers (Z-Neuro) illustrated that neuromarker concentrations in the lumbar CSF decreased over time in patients who survived, but increased in those who died (Figure 5A). For neuromarkers in lumbar CSF, PC1 + PC2 explained 86.6% of variability in the data over time; in ventricular CSF, PC1 + PC2 explained 92.5%. For the combined inflammatory markers (Z-Inflammatory), patients who died followed a similar downward trajectory to those who survived (Figure 5B); 52.5% of the variability was described by the first 2 PCs in lumbar CSF and 52.6% in ventricular CSF.

#### ROC Analysis

An increase in neuromarker concentrations from admission to week 2 predicted mortality with the best combination of sensitivity and specificity (Table 3); in particular, ΔS100B as little as 0.06 μg/L had 100% sensitivity and 93.1% specificity for predicting mortality. ROCs were not constructed for inflammatory markers due to the poor outcome association.

**Table 3.** Receiver Operating Characteristic and Outcome Analysis

Neuromarker Threshold	Sensitivity, %	Specificity, %	AUC (95% CI)
Mortality			
S100B >2.75 μg/L	71.43	70.97	0.69 (.4–.97)
NSE >56.84 μg/L	77.42	78.95	0.81 (.63–.99)
GFAP >27.25 μg/L	85.71	73.68	0.84 (.71–.98)
ΔS100B >0.06 μg/L	100	93.1	0.97 (.92–1)
ΔNSE >2.07 μg/L	85.71	65.52	0.81 (.56–1)
ΔGFAP >12.54 μg/L	85.7	96.5	0.87 (.64–1)
Morbidity			
S100B >2 μg/L	83.33	61.54	0.72 (.53–.9)
NSE >38.86 μg/L	75	65.38	0.77 (.62–.92)
GFAP >27.25 μg/L	50	65.38	0.69 (.51–.86)
ΔS100B >0.06 μg/L	66.67	95.83	0.73 (.51–.96)
ΔNSE >8.67 μg/L	58.33	91.67	0.75 (.54–.95)
ΔGFAP >12.54 μg/L	85.7	96.5	0.68 (.48–.88)

The table demonstrates concentrations of S100B, NSE, and GFAP and increases in their concentrations over the first 2 weeks (Δ), which predicted mortality and morbidity (dichotomized clinical outcome at 6 months) with the best combination of sensitivity and specificity.

Abbreviations: AUC, area under the curve; CI, confidence interval; GFAP, glial fibrillary acidic protein; NSE, neuron-specific enolase.

#### Multivariate Analysis

An increase between admission and week 2 in S100B, NSE, GFAP, and Z-Neuro all demonstrated associations with death in multiple logistic regression models (Table 4). The odds of death were 23 times greater with a 1-unit increase in Z-Neuro Δ (95% CI, 1.57–324.66; *P* = .02) and 9 times greater per-unit increase in ΔS100B (95% CI, 1.9–42.1, *P* = .005). Patients with an increase in ΔNSE and ΔGFAP had only slightly higher odds of death (95% CI, 1–1.1; *P* = .02) and (95% CI, 1–1.2; *P* = .01), respectively. Covariates included in the models were British Medical Research Council staging, symptom duration, age, sex, human immunodeficiency virus status, fever, seizures, culture positivity, hydrocephalus severity, and tuberculomas. Infarcts could not be added because they perfectly predicted death. Only the inclusion of seizures and tuberculomas altered these models (results shown in Table 3). Some of the multivariate models were also predictive of dichotomized clinical outcome at 6 months; the odds of a poor outcome were 4.1 times greater per-unit increase in Z-Neuro Δ (95% CI, 1.31–13.04, *P* = .02), and only slightly higher in patients with an increase in ΔNSE (95% CI, 1.01–1.11, *P* = .02). These relationships were not affected by clinical, admission, or radiologic characteristics.

**Table 4.** Multivariate Outcome Analysis

Variable	Outcome	Odds Ratio (95% CI)	*P* Value
Z-Neuro^a^ Δ	Mortality	22.5 (1.57–324.66)	.02
With seizure covariate added to model	Mortality	26.3 (1.8–383.05)	.02
	6-mo clinical outcome	4.1 (1.31–13.04)	.02
ΔS100B	Mortality	8.9 (1.9–42.1)	.005
	6-mo clinical outcome	1.9 (.96–3.65)	.06
ΔNSE	Mortality	1.07 (1–1.1)	.02
	6-mo clinical outcome	1.06 (1.01–1.11)	.02
ΔGFAP	Mortality	1.09 (1–1.2)	.01
With seizures added as a covariate to model		1.1 (1.02–1.21)	.01
With tuberculomas added as a covariate		0.5 (1.01–1.2)	.02
	6-mo clinical outcome	1.04 (.1–1.09)	.06

Abbreviations: Δ, change from admission to week 2 concentrations; CI, confidence interval; GFAP, glial fibrillary acidic protein; NSE, neuron-specific enolase.

^a^Z-Neuro represents a combined *z* score for all 3 neuromarkers.

## DISCUSSION

This study demonstrated the prolonged elevation of CSF S100B, NSE, and GFAP and their association with poor outcome, in particular when they increased over time. These markers may help elucidate the causes of progressive injury in these patients. Neither neuro- nor inflammatory markers were elevated in serum, suggesting that serum reflects little of the injury in the CNS, and that the immune response is compartmentalized to the site of disease. Elevated inflammatory markers in the CSF decreased in all patients regardless of outcome.

### Biomarker Elevations in TBM Cases

The highest CSF neuromarker concentrations were observed in the ventricular CSF, consistent with their cerebral origin. Lower lumbar concentrations were likely due to degradation by proteinases as CSF flows down the rostrocaudal gradient of the brain–spine axis [22–24]. However, cytokine concentrations were greater in lumbar CSF, possibly because much of the inflammatory process occurs in the subarachnoid space and because 76% of patients also had spinal disease. These differences are important in interpreting results from different compartments, especially where lumbar CSF results are reported without concurrent spinal imaging. Determining treatment/prognostic thresholds for biomarkers is challenging; studies demonstrate variability in biomarker concentrations across brain injury etiologies, testing platforms, and age of patients [15].

Serum neuromarker concentrations were low. Various factors may determine the concentration of brain-derived proteins in the blood, including their intrathecal concentration, response to pathology, diffusion across the blood–brain and blood–CSF barrier, and half-life in the blood. S100B, NSE, and GFAP are detectable in the blood early after traumatic brain injury or stroke [25–27]; however, their half-lives are estimated between 30 minutes and 48 hours [28, 29]. Consequently, serum concentrations likely reflect only a fraction of CSF concentrations and the timing of sampling relative to the onset of injury is important, but difficult to identify in a chronic condition like TBM where injury may also not be a single event. Episodes of ischemia and peaks in serum concentrations may, therefore, have been missed, especially if patients presented late.

### Association With Radiology

Neuromarker concentrations were not associated with spinal disease, possibly because neuromarkers of spinal origin may be masked by larger concentrations of cerebral origin. The association between severity of hydrocephalus and high GFAP and S100B concentrations suggests that these markers may be sensitive to transient injury due to the mechanical effect of dilating ventricles on the parenchyma, often accompanied by the opposing pressure from brain edema [30, 31]. The lack of significant differences in highest neuromarker concentrations between patients with and without infarcts may reflect the fact that 35% of patients with infarcts had only small lesions. Overall neuromarker concentrations were greater with large infarcts; their increasing profiles over time likely reflected ischemia-induced progressive injury. Because infarcts detected on imaging are already established, tracking these biomarkers could highlight patients at high risk of deterioration before irreversible events occur and increase surveillance and supportive measures (including supplemental oxygen, blood pressure augmentation, and more aggressive ICP control). In this way, the neuromarkers could complement imaging and other clinical measures of disease severity and progression. They may also help us better understand the pathophysiological process and the dynamic nature of the disease. Additionally, markers may also act as a proxy for disease response to intervention.

The association between high initial concentrations of ventricular CSF IFN-γ and TNF-α with mild enhancement and an absence of infarcts on admission scan may represent the early phases of the inflammatory process. In this cohort, tuberculomas and infarcts were negatively correlated, and tuberculomas were predictive of survival whereas infarcts were predictive of death [21]. This may represent the evolution of disease with tuberculomas representing an early phase of the disease that progresses to more severe disease with infarcts if treatment is delayed. Alternatively, it may be that the underlying pathophysiology leading to the development of tuberculomas is different from that leading to infarction, as demonstrated by the differential cytokine profiles. A localized T-helper 1 response promoted by IL-12p40 and reflected in elevated IP-10 and MCP-1 concentrations may promote tuberculoma formation, whereas elevated chemokines TNF-α, MIP-1α, IL-6, and IL-8 may provoke diffuse cellular infiltration and inflammation, contributing to more extensive infarction.

### Association With Clinical Outcome

Increasing neuromarker concentrations in CSF was the best indication of poor outcome, especially ΔS100B, suggesting that cerebral injury in TBM continues to evolve in some patients and leads to poor outcome despite the initiation of anti-TB and steroid treatment. The associated decreasing trend of inflammatory markers in patients who died suggests that it may be less an ongoing immune response that is responsible for the progressive brain injury associated with TBM, and more the secondary pathological processes that are initiated by the inflammatory process and continue to cause tissue injury. These processes may include biochemical and inflammatory mechanisms of secondary brain injury. This highlights the need for not only anti-TB and immunomodulatory drugs, but also better brain protection. More evidence of specific pathophysiological processes leading to an increase in neuromarkers is needed to direct brain management appropriately. If these processes are associated with brain ischemia, simple interventions such as more aggressive treatment of ICP, augmentation of blood pressure, and supplemental systemic oxygenation may improve oxygenation of the brain. Ongoing studies of pharmacological intervention in other forms of brain injury [32] may be applicable also in meningitis if there are similar underlying mechanisms.

Neuromarkers have predictive potential, either as point in time measures or, more particularly, over time. The profile over time may also be valuable in evaluating response to novel interventions. Ventricular CSF neuromarker concentrations appeared to be more predictive than lumbar CSF, likely because they arise from the predominant site of injury. In the resource-limited setting, assessment of injury may rely on a single marker; S100B is promising—an increase between admission and week 2 was associated with infarct severity and also strongly predicted mortality.

## LIMITATIONS

The sample size was modest and the age range too variable to adequately compare cases with age-matched controls; however, the role of age in normative biomarker concentrations remains contentious and there is no clear indication of how age influences normal ranges [15]. More frequent sampling during the acute phase (admission to week 2) may have provided greater insight into the dynamic nature of disease evolution. However, patients presented at different stages of the disease course and so peak concentrations may have been missed. The cytokines measured were not associated with injury severity or outcome; it may be that an unbiased exploration such as transcriptomic profiling would generate a better understanding of the association between the immune response and outcome. The complexity of the data set limited the statistical tools for examining outcome association, and correction for multiple comparisons for the association between the inflammatory markers and radiology was not conducted. PCA enabled combining multiple biomarkers over time but does not provide insight into the biology of the disease process; however, this study aimed to collect pilot data and generate hypotheses. This study was limited to patients with associated hydrocephalus and findings may not be generalizable to patients without hydrocephalus. Comparing neuromarker concentrations in TBM cases to a control group of patients with non-TB CNS infections would be valuable to discern the specificity of these neuromarkers and the range of elevation for TBM. This was beyond the scope of this pilot study and would be a valuable addition to a follow-up study.

## CONCLUSIONS

Neuromarkers in the CSF of patients with TBM provide insight into ongoing cerebral injury and prognosis. They may help direct limited resources and act as surrogate markers for the efficacy of novel interventions. Treatment or injury thresholds should be established for the ventricular and lumbar CSF compartments. Serum is uninformative for examining cerebral inflammation and injury. The peak of inflammation likely occurs early and decreases on treatment. Further examination of the secondary injury processes initiated by the inflammatory process is needed.

## Supplementary Data

Supplementary materials are available at *Clinical Infectious Diseases* online. Consisting of data provided by the authors to benefit the reader, the posted materials are not copyedited and are the sole responsibility of the authors, so questions or comments should be addressed to the corresponding author.

## Supplementary Material

Supplementary Material

## Notes

***Financial support.*** This work was supported by the Wellcome Trust (grant number 084323 to U. K. R.).

***Potential conflicts of interest.*** All authors: No reported conflicts of interest. All authors have submitted the ICMJE Form for Disclosure of Potential Conflicts of Interest. Conflicts that the editors consider relevant to the content of the manuscript have been disclosed.
