# Supplementary material for: Biomarkers of Cerebral Injury and Inflammation in Pediatric Tuberculous Meningitis
Source: Clin Infect Dis. 2017 Jun 9;65(8):1298–307. doi: 10.1093/cid/cix540 (PMC5815568; doi:10.1093/cid/cix540)
Supplement: Supplementary Material [file cix540_suppl_supplementary_material.docx]

**Supplementary material**

**Supplement 1: Biomarker analysis**

*Neuromarkers*

Commercially available enzyme-linked immunoassays (ELISA) were used to analyse S100B (Merck Millipore, Billerica, USA), NSE (DRG Diagnostics, Marburg, Germany) and GFAP (Merck Millipore, Billerica, USA). Batch analysis was performed thrice during the 3 years of patient enrolment based on the accumulation rate of samples. Testing procedures and ELISA kits were standard across the 3 analysis episodes. The full complement of each patient’s samples (serum and CSF) were analysed on the same plate. All assays were carried out according to manufacturer’s instructions. Testing kits for S100B and GFAP were validated for both serum and CSF. The NSE assay was validated for serum; however, since no NSE assays validated for CSF were available in South Africa, both sample types were analysed on this test kit. Normal reference ranges were suggested by the manufacturers for NSE only (0-12 ng/ml).

*Inflammatory markers*

The panel of cytokines and chemokines examined included IL-1β, IL-1Ra, IL-6, IL-10, IL-12p40, TNF-α, IFN-γ, IL-8, GRO, MCP-1, IP-10, MIP -1α, VEGF and RANTES. These were analysed on the Bio-Plex platform (Bio-Rad Laboratories, Hercules, CA, USA), using customised 14-plex MilliplexTM kits (Millipore, St Charles, MO, USA), according to the manufacturer’s instructions. The luminex plates were validated for serum and CSF in the Clinical Infectious Diseases and Research Initiative (CIDRI) laboratory.

**Data preparation**

Biomarker values that fell below the lowest limit of detection (LLOD) of the testing kit were assigned the value 0.01. Neuromarker values that exceeded the highest limit of detection (HLOD) and were extrapolated from the standard curve were accepted if the standard curve was linear, variability around the curve was very low (R^2^ = 0.98-0.99) and quality control values fell within the expected ranges. For cytokine concentrations that were so elevated that the luminex software did not extrapolate a value, the highest value measured for that analyte by that testing kit was assigned + 1.

**Sample exclusion criteria**

Biomarker data were reviewed in the clinical context of each patient. Seventeen samples were excluded for the following reasons: samples taken within 24-48 hours after procedures like insertion of external ventricular drains, endoscopic third ventriculostomies, CSF shunts and air encephalograms were identified and excluded to avoid the contribution of invasive procedures to inflammatory and injury marker concentrations. Samples that were not immediately refrigerated or not processed and frozen within 12 hours of collection were excluded as analyte degradation may have occurred. Hemolyzed or xanthochromic samples were excluded from NSE analysis to exclude NSE of erythrocyte origin.

**Supplement 2: PCA analysis**

Principal component analysis (PCA) was used as a data reduction technique to allow for the 2-dimensional visualization and simultaneous assessment of multiple biomarker values ($p$), taken in multiple sample types over time in a small sample size ^1^.

In PCA, the data are transformed to a set of $r<p$ principal components, which are (linear) combinations of the original variables that explain as much of the variation in the data as possible.

$$Z_{1}=w_{11}\times variable_{1}+w_{12}\times variable_{2}+w_{13}\times variable_{3}\ldots+w_{1p}\times variable_{p}$$

$$\vdots$$

$$Z_{r}=w_{r1}\times variable_{1}+w_{r2}\times variable_{2}+w_{r3}\times variable_{3}\ldots+w_{rp}\times variable_{p}$$

The coefficients ($w_{ij}, i=1\ldots r, j=1\ldots p)$ are called the principal component loadings. Substituting participant specific values for each of the original variables, we obtain a set of principal component scores (also known as Z-scores).

The first principal component is selected to represent as much of the variation in the original data as possible by a single component. The second principal component represents as much of the variation remaining after forming the first principal component and so forth. We can therefore define the quality of the PCA fit as a proportion of the total variance represented.

Plotting the first two principal component scores on a two dimensional co-ordinate system to represent the (n) sample points, together with information on the original variables, we obtain a biplot. This plot is interpreted in terms of distances and orthogonal projections to the axes.

For each of the sample types (lumbar and ventricular CSF and serum), PCA was performed using the admission neuro-and inflammatory marker data. The Z-scores for each participant for the first two principal components were plotted on a biplot, and markers were used to differentiate between the cases and the controls (Figure 2 and 3).

Similarly, PCA biplots were obtained for admission neuro- and inflammatory marker data for cases only, with markers representing each of the three sample types (Figure 4).

These scores were further used as a proxy for a single index of neurological injury or inflammation, incorporating the contribution of multiple neuro- or inflammatory markers respectively (Z-Neuro and Z-Inflammatory).

Finally, 3-dimensional PCA biplots were obtained for the lumbar CSF neuro-and inflammatory markers, incorporating time (Figure 8). Patients who died were indicated in black and those who survived were indicated in pink.

1 Helmy A, Antoniades CA, Guilfoyle MR, Carpenter KL, Hutchinson PJ. Principal component analysis of the cytokine and chemokine response to human traumatic brain injury. PLoS One 2012;7:e39677.

**Supplement 3: Radiological criteria**

Currently no standardized criteria exist to classify radiological characteristics like the severity of hydrocephalus or the size of infarcts. These criteria were determined by 3 senior pediatric radiologists and 1 senior pediatric neurosurgeon until consensus was reached. Reviewers were blind to patient outcome.

Brain imaging - *Hydrocephalus* was defined as mild (visible temporal horns, rounding of the third ventricle [V3]), moderate (all ventricles dilated, no transependymal fluid shift) or severe (dilated ventricles, fluid shift and loss of sulcal markings). *Basal enhancement* was classified as focal (localized enhancement) or diffuse (enhancement not limited to a focal region). *Infarct*s were recorded as unilateral or bilateral, and their size was graded as single (lacunar), multiple/large (multiple or large branch infarcts) and large territory (encompassing MCA, ACA, PCA territories). *Tuberculomas* were classified by number.

Spinal imaging (MRI with contrast) - *Arachnoiditis* was defined as enhancement of the spinal cord or roots; with nodules, exudate and clumping of nerve roots, and the presence of plaques (extra or intra-dural collections of exudate) noted. *Tuberculoma or TB abscesses* were categorized as surface or intramedullary.

Some examples of these radiological characteristics are included below:

**Hydrocephalus**


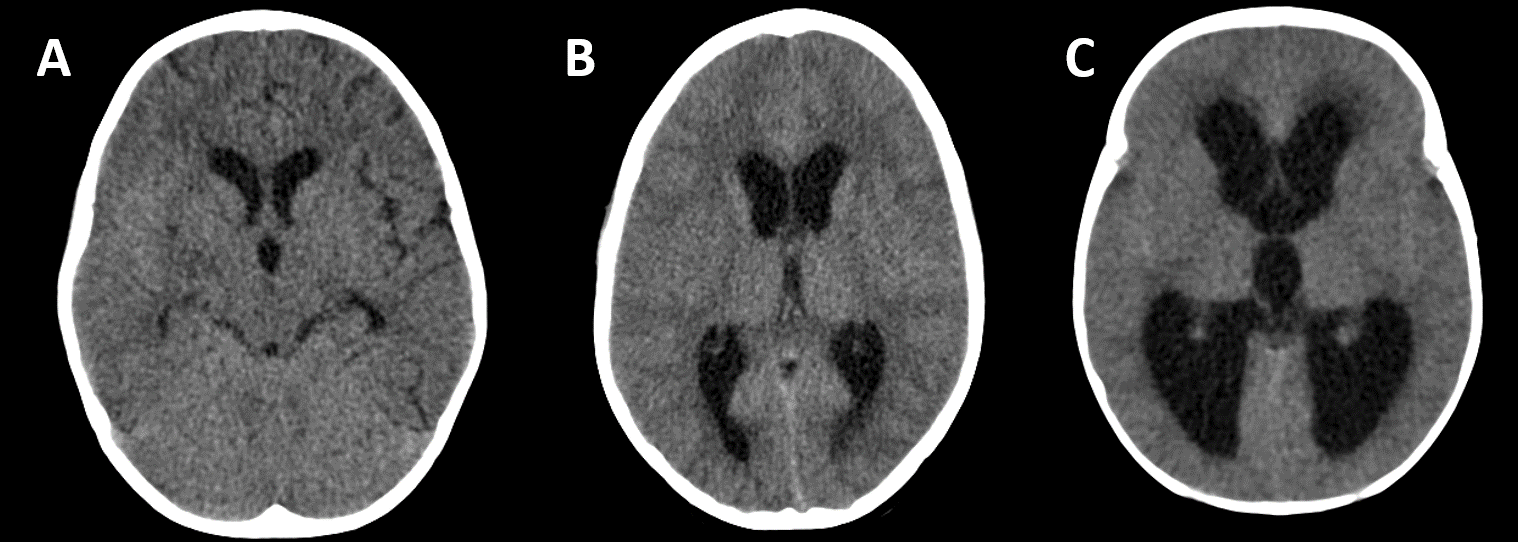


Axial uncontrasted CT brain scans in patients with TBM showing (A) mild hydrocephalus with a rounded 3^rd^ ventricle, (B) moderate hydrocephalus with ventricular dilatation but no transependymal fluid shift, (C) severe hydrocephalus with diffuse ventricular dilatation and transependymal fluid shift.

**Infarcts**


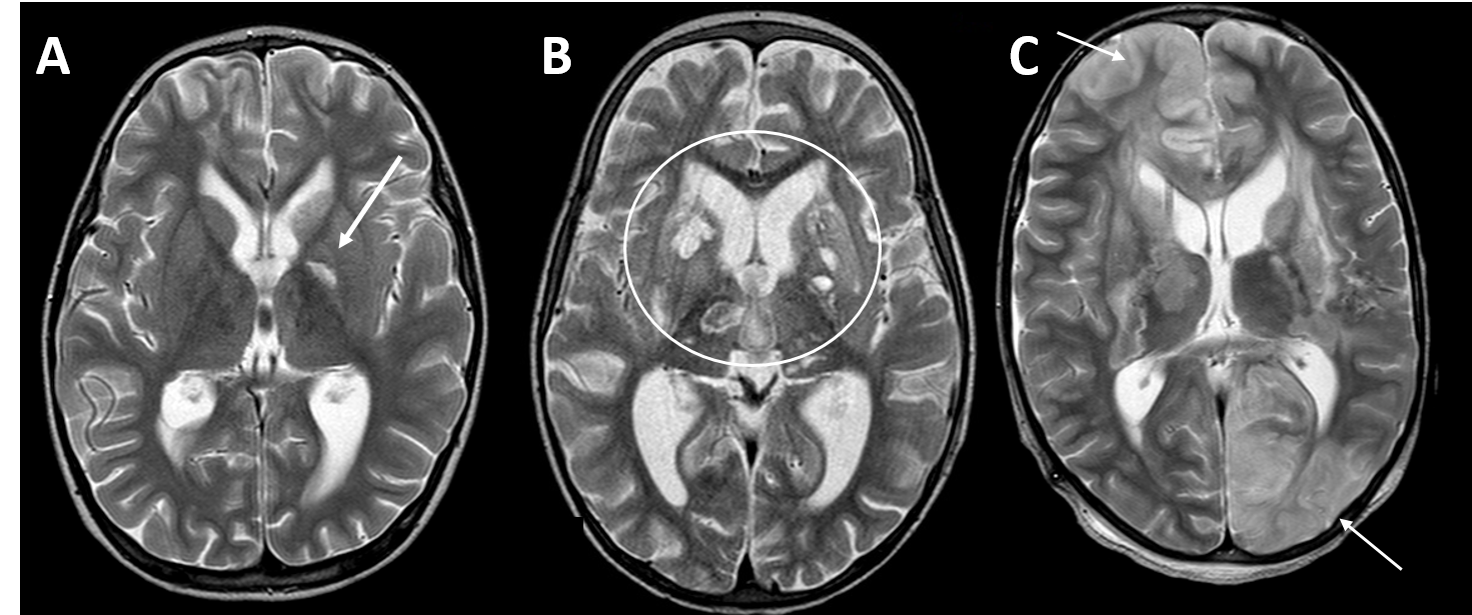


Axial T2-weighted MRIs in patients with TBM showing (A) small focal infarct involving the basal ganglia (arrow), (B) multiple bilateral basal ganglia infarcts (circled), (C) multiple hemispheric (arrows) and basal ganglia infarcts.

**Tuberculoma**

Axial post-contrast CT brain scan showing a peripherally enhancing tuberculoma (arrow) in a TBM patient


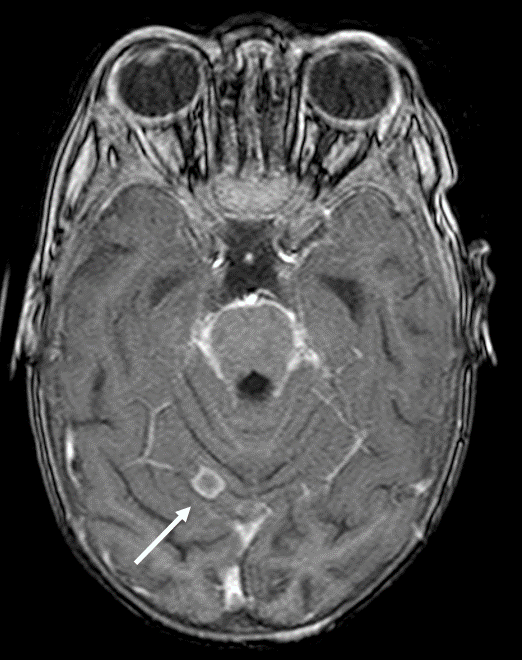


**Spinal disease**

Sagittal T1 weighted MRI postcontrast in a TBM patient showing (1) diffuse enhancement surrounding the cord, (2) enhancing extramedullary exudate plaque-like collection, (3) diffuse hyperintense exudate filling the thecal sac


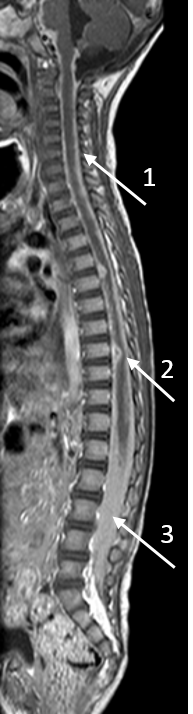


**Supplement 4: Biomarker analysis results and descriptive statistics**

A total of 130 lumbar CSF, 46 ventricular CSF and 107 serum samples were analyzed from TBM cases, 17 samples were excluded (n=7 lumbar CSF, 4 ventricular CSF, 6 serum) because samples were taken within 24-48 hours after neurosurgical procedures or were not frozen within 12 hours. Hemolyzed serum samples (n=15) were excluded. An additional lumbar CSF and serum sample were excluded from Luminex analysis due to a technical error. Eleven lumbar CSF and serum sample pairs from fatty filum controls, and 9 serum samples from pTB controls were analysed. Hemolyzed samples were excluded from NSE analysis (n=1 per control group).

*Assay results*

All quality control values in the ELISA and Luminex assays fell within the expected ranges. All standard curves generated in the ELISA results had R^2^ values between 0.98 and 0.99. Descriptive statistics for neuro- and inflammatory marker concentrations for cases and controls, and the results of the Mann-Whitney’s U comparison are tabulated below.

| **Marker** | | **S100B Cases (ug/L)** | | **S100B controls (ug/L)** | **Cases vs controls**  **Lumbar** | **Cases vs controls**  **Vent** | **S100B Cases (ug/L)** | **Fatty filum controls (ug/L)** | **pTB controls (ug/L)** | **Cases vs fatty filum controls** | **Cases vs pTB controls** |
| --- | --- | --- | --- | --- | --- | --- | --- | --- | --- | --- | --- |
| **Sample Type** | | **Lumbar** | **Vent** | **Lumbar** | **p-value** | **p-value** | **Serum** | **Serum** | **Serum** | **p-value** | **p-value** |
| **Admission** | | n=36 | n=23 | n=11 | 0.002* | 0.002* | n=36 | n=11 | n=9 | 1 | 1 |
|  | Median | 1.37 | 3.24 | 0.36 |  |  | 0.08 | 0.05 | 0.1 |  |  |
|  | Min | 0.01 | 0.91 | 0.01 |  |  | 0.01 | 0.03 | 0.01 |  |  |
|  | Max | 8.57 | 9.82 | 0.44 |  |  | 0.59 | 0.15 | 0.5 |  |  |
|  | p25 | 0.7 | 1.34 | 0.21 |  |  | 0.02 | 0.04 | 0.07 |  |  |
|  | p75 | 3.07 | 5.30 | 0.39 |  |  | 0.14 | 0.1 | 0.11 |  |  |
|  | p95 |  |  | 0.44 |  |  |  | 0.15 | 0.5 |  |  |
| **Week 1** | | n=29 | n=9 |  | 0.003* | 0.003* | n=26 |  |  | 1 | 1 |
|  | Median | 1.37 | 5.37 |  |  |  | 0.11 |  |  |  |  |
|  | Min | 0.16 | 1.07 |  |  |  | 0.01 |  |  |  |  |
|  | Max | 9.13 | 16.25 |  |  |  | 1.00 |  |  |  |  |
|  | p25 | 0.57 | 1.13 |  |  |  | 0.02 |  |  |  |  |
|  | p75 | 3.46 | 9.12 |  |  |  | 0.23 |  |  |  |  |
| **Week 2** | | n=31 | n=6 |  | 0.01* | 0.09 | n=24 |  |  | 1 | 1 |
|  | Median | 0.77 | 1.05 |  |  |  | 0.04 |  |  |  |  |
|  | Min | 0.16 | 0.29 |  |  |  | 0.01 |  |  |  |  |
|  | Max | 10.53 | 2.53 |  |  |  | 1.06 |  |  |  |  |
|  | p25 | 0.4 | 0.62 |  |  |  | 0.03 |  |  |  |  |
|  | p75 | 1.84 | 2.47 |  |  |  | 0.22 |  |  |  |  |
| **Week 3** | | n=21 | n=5 |  | 0.43 | 0.28 | n=14 |  |  | 1 | 1 |
|  | Median | 0.48 | 1.10 |  |  |  | 0.06 |  |  |  |  |
|  | Min | 0.01 | 0.12 |  |  |  | 0.01 |  |  |  |  |
|  | Max | 16.9 | 11.86 |  |  |  | 0.44 |  |  |  |  |
|  | p25 | 0.22 | 0.86 |  |  |  | 0.03 |  |  |  |  |
|  | p75 | 1.01 | 3.81 |  |  |  | 0.11 |  |  |  |  |
| **Week 4** | | n=11 |  |  | 0.4 |  | n=7 |  |  | 1 | 1 |
|  | Median | 0.62 |  |  |  |  | 0.07 |  |  |  |  |
|  | Min | 0.01 |  |  |  |  | 0.03 |  |  |  |  |
|  | Max | 1.41 |  |  |  |  | 0.15 |  |  |  |  |
|  | p25 | 0.03 |  |  |  |  | 0.05 |  |  |  |  |
|  | p75 | 0.83 |  |  |  |  | 0.12 |  |  |  |  |

Lumbar = lumbar CSF, Vent = ventricular CSF, p= percentile, min = minimum, max = maximum, pTB = pulmonary TB controls, * = statistically significant at *p<0.05,* only 2 patients had ventricular CSF samples taken in week 4, due the low sample size biomarker concentrations are not presented

| **Marker** | | **NSE Cases (ug/L)** | | **NSE controls (ug/L)** | **Cases vs controls**  **Lumbar** | **Cases vs controls**  **Vent** | **NSE Cases (ug/L)** | **Fatty filum controls (ug/L)** | **pTB controls (ug/L)** | **Cases vs fatty filum controls** | **Cases vs pTB controls** |
| --- | --- | --- | --- | --- | --- | --- | --- | --- | --- | --- | --- |
| **Sample Type** | | **Lumbar** | **Vent** | **Lumbar** | **p-value** | **p-value** | **Serum** | **Serum** | **Serum** | **p-value** | **p-value** |
| **Admission** | | n=36 | n=23 | n=10 | 0.01* | 0.003* | n=30 | n=10 | n=7 | 1 | 1 |
|  | Median | 17.62 | 29.07 | 6.88 |  |  | 16.74 | 13.4 | 28.12 |  |  |
|  | Min | 0.01 | 0.01 | 2.87 |  |  | 0.01 | 8.2 | 10.42 |  |  |
|  | Max | 89.49 | 104.86 | 12.46 |  |  | 73.75 | 26.23 | 50.82 |  |  |
|  | p25 | 8.48 | 20.53 | 4.51 |  |  | 11.36 | 10.9 | 13.39 |  |  |
|  | p75 | 38.12 | 48.89 | 10.42 |  |  | 27.08 | 20.9 | 29.34 |  |  |
|  | p95 |  |  | 12.46 |  |  |  | 26.23 | 50.82 |  |  |
| **Week 1** | | n=29 | n=9 |  | 0.01* | 0.003* | n=21 |  |  | 1 | 1 |
|  | Median | 24.67 | 39.61 |  |  |  | 16.09 |  |  |  |  |
|  | Min | 0.01 | 17.38 |  |  |  | 0.01 |  |  |  |  |
|  | Max | 94.97 | 154.86 |  |  |  | 42.60 |  |  |  |  |
|  | p25 | 14.32 | 34.61 |  |  |  | 8.8 |  |  |  |  |
|  | p75 | 46.42 | 91.97 |  |  |  | 24.21 |  |  |  |  |
| **Week 2** | | n=31 | n=6 |  | 0.06 | 0.28 | n=23 |  |  | 1 | 0.79 |
|  | Median | 14.44 | 19.42 |  |  |  | 12.86 |  |  |  |  |
|  | Min | 0.01 | 0.01 |  |  |  | 0.01 |  |  |  |  |
|  | Max | 78.92 | 80.64 |  |  |  | 104.67 |  |  |  |  |
|  | p25 | 8.21 | 10.82 |  |  |  | 7.99 |  |  |  |  |
|  | p75 | 28.8 | 46.01 |  |  |  | 19.88 |  |  |  |  |
| **Week 3** | | n=21 | n=5 |  | 0.43 | 0.28 | n=13 |  |  | 1 | 0.1 |
|  | Median | 10.52 | 29.20 |  |  |  | 12.74 |  |  |  |  |
|  | Min | 0.01 | 0.01 |  |  |  | 5.82 |  |  |  |  |
|  | Max | 101.92 | 44.64 |  |  |  | 50.08 |  |  |  |  |
|  | p25 | 8.75 | 13.86 |  |  |  | 7.66 |  |  |  |  |
|  | p75 | 20.08 | 34.52 |  |  |  | 18.62 |  |  |  |  |
| **Week 4** | | n=11 |  |  | 0.46 | 0.24 | n=5 |  |  | 1 | 1 |
|  | Median | 7.54 |  |  |  |  | 16.28 |  |  |  |  |
|  | Min | 0.01 |  |  |  |  | 0.01 |  |  |  |  |
|  | Max | 44.34 |  |  |  |  | 32.70 |  |  |  |  |
|  | p25 | 6.51 |  |  |  |  | 14.07 |  |  |  |  |
|  | p75 | 18.34 |  |  |  |  | 22.45 |  |  |  |  |

Lumbar = lumbar CSF, Vent = ventricular CSF, p= percentile, min = minimum, max = maximum, pTB = pulmonary TB controls, * = statistically significant at *p<0.05*, only 2 patients had ventricular CSF samples taken in week 4, due the low sample size biomarker concentrations are not presented

| **Marker** | | **GFAP Cases (ug/L)** | | **GFAP controls (ug/L)** | **Cases vs controls**  **Lumbar** | **Cases vs controls**  **Vent** | **GFAP Cases (ug/L)** | **Fatty filum controls (ug/L)** | **pTB controls (ug/L)** | **Cases vs fatty filum controls** | **Cases vs pTB controls** |
| --- | --- | --- | --- | --- | --- | --- | --- | --- | --- | --- | --- |
| **Sample Type** | | **Lumbar** | **Vent** | **Lumbar** | **p-value** | **p-value** | **Serum** | **Serum** | **Serum** | **p-value** | **p-value** |
| **Admission** | | n=36 | n=23 | n=11 | 0.002* | 0.002* | n=36 | n=11 | n=9 | 0.68 | 1 |
|  | Median | 8.92 | 93.31 | 0.03 |  |  | 0.01 | 0.76 | 1.17 |  |  |
|  | Min | 0.01 | 2.73 | 0 |  |  | 0.01 | 0.01 | 0.01 |  |  |
|  | Max | 181.09 | 269.96 | 1.19 |  |  | 20.25 | 5.90 | 5.90 |  |  |
|  | p25 | 3.25 | 33.86 | 0 |  |  | 0.02 | 0.24 | 0.01 |  |  |
|  | p75 | 24.16 | 172.37 | 0.62 |  |  | 1.77 | 2.51 | 1.79 |  |  |
|  | p95 |  |  | 1.19 |  |  |  | 5.9 | 5.79 |  |  |
| **Week 1** | | n=29 | n=9 |  | 0.002* | 0.003* | n=26 |  |  | 1 | 1 |
|  | Median | 6.06 | 168.64 |  |  |  | 0.01 |  |  |  |  |
|  | Min | 0.01 | 8.23 |  |  |  | 0.01 |  |  |  |  |
|  | Max | 298.66 | 344.40 |  |  |  | 16.29 |  |  |  |  |
|  | p25 | 2.1 | 13.37 |  |  |  | 0.01 |  |  |  |  |
|  | p75 | 21.84 | 331.87 |  |  |  | 0.01 |  |  |  |  |
| **Week 2** | | n=31 | n=6 |  | 0.01* | 0.07 | n=24 |  |  | 1 | 1 |
|  | Median | 3.38 | 66.30 |  |  |  | 0.01 |  |  |  |  |
|  | Min | 0.01 | 0.01 |  |  |  | 0.01 |  |  |  |  |
|  | Max | 164.43 | 270.50 |  |  |  | 15.88 |  |  |  |  |
|  | p25 | 0.03 | 17.09 |  |  |  | 0.01 |  |  |  |  |
|  | p75 | 9.38 | 100.01 |  |  |  | 2.72 |  |  |  |  |
| **Week 3** | | n=21 | n=5 |  | 0.002* | 0.02* | n=14 |  |  | 1 | 1 |
|  | Median | 3.26 | 196.12 |  |  |  | 0.89 |  |  |  |  |
|  | Min | 0.01 | 18.55 |  |  |  | 0.01 |  |  |  |  |
|  | Max | 257.8 | 281.20 |  |  |  | 20.79 |  |  |  |  |
|  | p25 | 0.67 | 62.29 |  |  |  | 0.01 |  |  |  |  |
|  | p75 | 10.27 | 271.99 |  |  |  | 2.21 |  |  |  |  |
| **Week 4** | | n=11 |  |  | 0.01* | 0.23 | n=7 |  |  | 1 | 1 |
|  | Median | 3.46 |  |  |  |  | 0.01 |  |  |  |  |
|  | Min | 0.01 |  |  |  |  | 0.01 |  |  |  |  |
|  | Max | 30.64 |  |  |  |  | 31.72 |  |  |  |  |
|  | p25 | 1.62 |  |  |  |  | 0.01 |  |  |  |  |
|  | p75 | 9.4 |  |  |  |  | 1.4 |  |  |  |  |

Lumbar = lumbar CSF, Vent = ventricular CSF, p= percentile, min = minimum, max = maximum, pTB = pulmonary TB controls, * = statistically significant at *p<0.05,* only 2 patients had ventricular CSF samples taken in week 4, due the low sample size biomarker concentrations are not presented

| **Marker** | | **TNF-α Cases (pg/mL)** | | **TNF-α controls (pg/mL)** | **Cases vs controls**  **Lumbar** | **Cases vs controls**  **Vent** | **TNF-α Cases (pg/mL)** | **Fatty filum controls (pg/mL)** | **pTB controls (pg/mL)** | **Cases vs fatty filum controls** | **Cases vs pTB controls** |
| --- | --- | --- | --- | --- | --- | --- | --- | --- | --- | --- | --- |
| **Sample Type** | | **Lumbar** | **Vent** | **Lumbar** | **p-value** | **p-value** | **Serum** | **Serum** | **Serum** | **p-value** | **p-value** |
| **Admission** | | n=36 | n=23 | n=11 | p<0.01* | p<0.01* | n=36 | n=11 | n=9 | p=0.78 | p=0.01* |
|  | Median | 266.32 | 109 | 0.01 |  |  | 15.46 | 12.09 | 27.4 |  |  |
|  | Min | 76.97 | 20.63 | 0.01 |  |  | 0.01 | 7.7 | 11.84 |  |  |
|  | Max | 1604.31 | 1010.17 | 0.01 |  |  | 50.88 | 27.82 | 135.02 |  |  |
|  | p25 | 180.14 | 75.49 | 0.01 |  |  | 8.42 | 9.75 | 23.16 |  |  |
|  | p75 | 493.68 | 171.6 | 0.01 |  |  | 19.11 | 20.84 | 33.06 |  |  |
|  | p95 |  |  | 0.01 |  |  |  | 27.82 | 135.02 |  |  |
| **Week 1** | | n=29 | n=9 |  |  |  | n=26 |  |  |  |  |
|  | Median | 160.16 | 39.89 |  |  |  | 13.48 |  |  |  |  |
|  | Min | 34.63 | 12.09 |  |  |  | 3.36 |  |  |  |  |
|  | Max | 1329.57 | 712.6 |  |  |  | 52.55 |  |  |  |  |
|  | p25 | 83.49 | 32.65 |  |  |  | 8.86 |  |  |  |  |
|  | p75 | 295.86 | 74.18 |  |  |  | 17.94 |  |  |  |  |
| **Week 2** | | n=30 | n=6 |  |  |  | n=23 |  |  |  |  |
|  | Median | 95.15 | 67.55 |  |  |  | 11.45 |  |  |  |  |
|  | Min | 25.48 | 15.8 |  |  |  | 3.99 |  |  |  |  |
|  | Max | 402.75 | 141.27 |  |  |  | 25.31 |  |  |  |  |
|  | p25 | 48.62 | 50.58 |  |  |  | 7.19 |  |  |  |  |
|  | p75 | 138.29 | 137.06 |  |  |  | 14.94 |  |  |  |  |
| **Week 3** | | n=21 | n=5 |  |  |  | n=14 |  |  |  |  |
|  | Median | 56.84 | 90.7 |  |  |  | 11.7 |  |  |  |  |
|  | Min | 8.49 | 17.74 |  |  |  | 4.12 |  |  |  |  |
|  | Max | 216.71 | 118.5 |  |  |  | 50.26 |  |  |  |  |
|  | p25 | 26.16 | 29.96 |  |  |  | 5.52 |  |  |  |  |
|  | p75 | 84 | 110.26 |  |  |  | 16.64 |  |  |  |  |
| **Week 4** | | n=11 |  |  |  |  | n=7 |  |  |  |  |
|  | Median | 56.05 |  |  |  |  | 13.8 |  |  |  |  |
|  | Min | 20.5 |  |  |  |  | 5.35 |  |  |  |  |
|  | Max | 299.88 |  |  |  |  | 39.14 |  |  |  |  |
|  | p25 | 34.94 |  |  |  |  | 6.48 |  |  |  |  |
|  | p75 | 129.88 |  |  |  |  | 29.52 |  |  |  |  |

Lumbar = lumbar CSF, Vent = ventricular CSF, p= percentile, min = minimum, max = maximum, pTB = pulmonary TB controls, * = statistically significant at *p<0.05,* lumbar and serum samples in week 2 have 1 less sample relative to neuromarkers as this pair of samples was excluded due to a technical error during Luminex analysis, only 2 patients had ventricular CSF samples taken in week 4, due the low sample size biomarker concentrations are not presented

| **Marker** | | **IFN-γ Cases (pg/mL)** | | **IFN-γ controls (pg/mL)** | **Cases vs controls**  **Lumbar** | **Cases vs controls**  **Vent** | **IFN-γ Cases (pg/mL)** | **Fatty filum controls (pg/mL)** | **pTB controls (pg/mL)** | **Cases vs fatty filum controls** | **Cases vs pTB controls** |
| --- | --- | --- | --- | --- | --- | --- | --- | --- | --- | --- | --- |
| **Sample Type** | | **Lumbar** | **Vent** | **Lumbar** | **p-value** | **p-value** | **Serum** | **Serum** | **Serum** | **p-value** | **p-value** |
| **Admission** | | n=36 | n=23 | n=11 | p<0.01* | p<0.01* | n=36 | n=11 | n=9 | p=0.02* | p=0.08 |
|  | Median | 1095.67 | 684.79 | 0.01 |  |  | 9.66 | 12.09 | 27.4 |  |  |
|  | Min | 140.99 | 118.89 | 0.01 |  |  | 0.01 | 7.7 | 11.84 |  |  |
|  | Max | 8541.02 | 3888.62 | 0.01 |  |  | 48.9 | 27.82 | 135.02 |  |  |
|  | p25 | 619.97 | 307.39 | 0.01 |  |  | 4.12 | 9.75 | 23.16 |  |  |
|  | p75 | 2809.42 | 1080.8 | 0.01 |  |  | 19.58 | 20.84 | 33.06 |  |  |
|  | p95 |  |  | 0.01 |  |  |  | 27.82 | 135.02 |  |  |
| **Week 1** | | n=29 | n=9 |  |  |  | n=26 |  |  |  |  |
|  | Median | 278.44 | 239.02 |  |  |  | 6.07 |  |  |  |  |
|  | Min | 48.44 | 57.14 |  |  |  | 0.01 |  |  |  |  |
|  | Max | 10275.4 | 1055.49 |  |  |  | 75.84 |  |  |  |  |
|  | p25 | 149.23 | 112.76 |  |  |  | 0.01 |  |  |  |  |
|  | p75 | 793.26 | 259.64 |  |  |  | 13.05 |  |  |  |  |
| **Week 2** | | n=30 | n=6 |  |  |  | n=23 |  |  |  |  |
|  | Median | 244.51 | 271.32 |  |  |  | 4.43 |  |  |  |  |
|  | Min | 0.01 | 25.92 |  |  |  | 0.01 |  |  |  |  |
|  | Max | 2197.81 | 787.56 |  |  |  | 44.78 |  |  |  |  |
|  | p25 | 80.71 | 108.88 |  |  |  | 0.01 |  |  |  |  |
|  | p75 | 465.49 | 507.03 |  |  |  | 13.57 |  |  |  |  |
| **Week 3** | | n=21 | n=5 |  |  |  | n=14 |  |  |  |  |
|  | Median | 126.68 | 93.92 |  |  |  | 0.01 |  |  |  |  |
|  | Min | 0.01 | 31.33 |  |  |  | 0.01 |  |  |  |  |
|  | Max | 757.17 | 315.17 |  |  |  | 268.25 |  |  |  |  |
|  | p25 | 66.08 | 67.74 |  |  |  | 0.01 |  |  |  |  |
|  | p75 | 154.25 | 184.12 |  |  |  | 7.04 |  |  |  |  |
| **Week 4** | | n=11 |  |  |  |  | n=7 |  |  |  |  |
|  | Median | 69.71 |  |  |  |  | 0.01 |  |  |  |  |
|  | Min | 21.1 |  |  |  |  | 0.01 |  |  |  |  |
|  | Max | 715.18 |  |  |  |  | 116.18 |  |  |  |  |
|  | p25 | 49.34 |  |  |  |  | 0.01 |  |  |  |  |
|  | p75 | 209.65 |  |  |  |  | 9.15 |  |  |  |  |

Lumbar = lumbar CSF, Vent = ventricular CSF, p= percentile, min = minimum, max = maximum, pTB = pulmonary TB controls, * = statistically significant at *p<0.05,* lumbar and serum samples in week 2 have 1 less sample relative to neuromarkers as this pair of samples was excluded due to a technical error during Luminex analysis, only 2 patients had ventricular CSF samples taken in week 4, due the low sample size biomarker concentrations are not presented

| **Marker** | | **IL-6 Cases (pg/mL)** | | **IL-6 controls (pg/mL)** | **Cases vs controls**  **Lumbar** | **Cases vs controls**  **Vent** | **IL-6 Cases (pg/mL)** | **Fatty filum controls (pg/mL)** | **pTB controls (pg/mL)** | **Cases vs fatty filum controls** | **Cases vs pTB controls** |
| --- | --- | --- | --- | --- | --- | --- | --- | --- | --- | --- | --- |
| **Sample Type** | | **Lumbar** | **Vent** | **Lumbar** | **p-value** | **p-value** | **Serum** | **Serum** | **Serum** | **p-value** | **p-value** |
| **Admission** | | n=36 | n=23 | n=11 | p<0.01* | p<0.01* | n=36 | n=11 | n=9 | p<0.01* | p=0.04* |
|  | Median | 1700.91 | 873.54 | 0.01 |  |  | 7.61 | 0.01 | 34.39 |  |  |
|  | Min | 218.79 | 38.35 | 0.01 |  |  | 49.95 | 0.01 | 3.82 |  |  |
|  | Max | 18448.02 | 13643.62 | 0.01 |  |  | 10.20 | 47.63 | 458.64 |  |  |
|  | p25 | 1101.84 | 415.62 | 0.01 |  |  | 1.96 | 0.01 | 7.21 |  |  |
|  | p75 | 4863.87 | 2272.35 | 0.01 |  |  | 12.35 | 0.01 | 40.48 |  |  |
|  | p95 |  |  | 0.01 |  |  |  | 47.63 | 458.64 |  |  |
| **Week 1** | | n=29 | n=9 |  |  |  | n=26 |  |  |  |  |
|  | Median | 595.86 | 377.59 |  |  |  | 4.73 |  |  |  |  |
|  | Min | 0.01 | 43.08 |  |  |  | 0.01 |  |  |  |  |
|  | Max | 10464.67 | 5174.25 |  |  |  | 392.83 |  |  |  |  |
|  | p25 | 328.33 | 58.56 |  |  |  | 0.01 |  |  |  |  |
|  | p75 | 2066.4 | 791.99 |  |  |  | 12.43 |  |  |  |  |
| **Week 2** | | n=30 | n=6 |  |  |  | n=23 |  |  |  |  |
|  | Median | 373.81 | 343.71 |  |  |  | 0.01 |  |  |  |  |
|  | Min | 21.68 | 18.89 |  |  |  | 0.01 |  |  |  |  |
|  | Max | 5175.25 | 1210.65 |  |  |  | 46.97 |  |  |  |  |
|  | p25 | 91.83 | 170.51 |  |  |  | 0.01 |  |  |  |  |
|  | p75 | 604.53 | 500.6 |  |  |  | 5.73 |  |  |  |  |
| **Week 3** | | n=21 | n=5 |  |  |  | n=14 |  |  |  |  |
|  | Median | 351.6 | 244.88 |  |  |  | 2.78 |  |  |  |  |
|  | Min | 5.65 | 5.22 |  |  |  | 0.01 |  |  |  |  |
|  | Max | 1586.25 | 21146.37 |  |  |  | 67.69 |  |  |  |  |
|  | p25 | 120.81 | 59.89 |  |  |  | 0.01 |  |  |  |  |
|  | p75 | 482.87 | 561.8 |  |  |  | 4.74 |  |  |  |  |
| **Week 4** | | n=11 |  |  |  |  | n=7 |  |  |  |  |
|  | Median | 216.2 |  |  |  |  | 0.01 |  |  |  |  |
|  | Min | 12.87 |  |  |  |  | 0.01 |  |  |  |  |
|  | Max | 1117.09 |  |  |  |  | 35.62 |  |  |  |  |
|  | p25 | 62.02 |  |  |  |  | 0.01 |  |  |  |  |
|  | p75 | 678.55 |  |  |  |  | 6.8 |  |  |  |  |

Lumbar = lumbar CSF, Vent = ventricular CSF, p= percentile, min = minimum, max = maximum, pTB = pulmonary TB controls, * = statistically significant at *p<0.05,* lumbar and serum samples in week 2 have 1 less sample relative to neuromarkers as this pair of samples was excluded due to a technical error during Luminex analysis, only 2 patients had ventricular CSF samples taken in week 4, due the low sample size biomarker concentrations are not presented

| **Marker** | | **IL-12p40 Cases (pg/mL)** | | **IL-12p40 controls (pg/mL)** | **Cases vs controls**  **Lumbar** | **Cases vs controls**  **Vent** | **IL-12p40 Cases (pg/mL)** | **Fatty filum controls (pg/mL)** | **pTB controls (pg/mL)** | **Cases vs fatty filum controls** | **Cases vs pTB controls** |
| --- | --- | --- | --- | --- | --- | --- | --- | --- | --- | --- | --- |
| **Sample Type** | | **Lumbar** | **Vent** | **Lumbar** | **p-value** | **p-value** | **Serum** | **Serum** | **Serum** | **p-value** | **p-value** |
| **Admission** | | n=36 | n=23 | n=11 | p<0.01* | p=0.01* | n=36 | n=11 | n=9 | p=0.4 | p=0.54 |
|  | Median | 43.34 | 4.85 | 0.01 |  |  | 10.69 | 29.25 | 25.69 |  |  |
|  | Min | 0.01 | 0.01 | 0.01 |  |  | 0.01 | 0.01 | 0.01 |  |  |
|  | Max | 279.46 | 63.99 | 0.01 |  |  | 126.18 | 99.76 | 74.31 |  |  |
|  | p25 | 20.25 | 0.01 | 0.01 |  |  | 0.01 | 5.74 | 6.51 |  |  |
|  | p75 | 62.8 | 32.03 | 0.01 |  |  | 45.2 | 41.58 | 35.11 |  |  |
|  | p95 |  |  | 0.01 |  |  |  | 99.76 | 74.31 |  |  |
| **Week 1** | | n=29 | n=9 |  |  |  | n=26 |  |  |  |  |
|  | Median | 44.96 | 2.39 |  |  |  | 17.29 |  |  |  |  |
|  | Min | 0.01 | 0.01 |  |  |  | 0.01 |  |  |  |  |
|  | Max | 330.96 | 114.41 |  |  |  | 114.21 |  |  |  |  |
|  | p25 | 25.2 | 0.01 |  |  |  | 0.01 |  |  |  |  |
|  | p75 | 79.95 | 12.54 |  |  |  | 38.13 |  |  |  |  |
| **Week 2** | | n=30 | n=6 |  |  |  | n=23 |  |  |  |  |
|  | Median | 50.99 | 23.7 |  |  |  | 21.47 |  |  |  |  |
|  | Min | 0.01 | 0.01 |  |  |  | 0.01 |  |  |  |  |
|  | Max | 356.42 | 133.38 |  |  |  | 92.16 |  |  |  |  |
|  | p25 | 30.99 | 16.74 |  |  |  | 0.01 |  |  |  |  |
|  | p75 | 100.02 | 35.15 |  |  |  | 44.04 |  |  |  |  |
| **Week 3** | | n=21 | n=5 |  |  |  | n=14 |  |  |  |  |
|  | Median | 41.37 | 64.48 |  |  |  | 13.99 |  |  |  |  |
|  | Min | 0.01 | 8.08 |  |  |  | 0.01 |  |  |  |  |
|  | Max | 217.54 | 146.87 |  |  |  | 236.27 |  |  |  |  |
|  | p25 | 4.87 | 12.89 |  |  |  | 0.01 |  |  |  |  |
|  | p75 | 65.95 | 87.23 |  |  |  | 23.44 |  |  |  |  |
| **Week 4** | | n=11 |  |  |  |  | n=7 |  |  |  |  |
|  | Median | 30.99 |  |  |  |  | 0.01 |  |  |  |  |
|  | Min | 0.01 |  |  |  |  | 0.01 |  |  |  |  |
|  | Max | 273.4 |  |  |  |  | 176.57 |  |  |  |  |
|  | p25 | 25.2 |  |  |  |  | 0.01 |  |  |  |  |
|  | p75 | 59.27 |  |  |  |  | 52.47 |  |  |  |  |

Lumbar = lumbar CSF, Vent = ventricular CSF, p= percentile, min = minimum, max = maximum, pTB = pulmonary TB controls, * = statistically significant at *p<0.05,* lumbar and serum samples in week 2 have 1 less sample relative to neuromarkers as this pair of samples was excluded due to a technical error during Luminex analysis, only 2 patients had ventricular CSF samples taken in week 4, due the low sample size biomarker concentrations are not presented

| **Marker** | | **IL-1β Cases (pg/mL)** | | **IL-1β controls (pg/mL)** | **Cases vs controls**  **Lumbar** | **Cases vs controls**  **Vent** | **IL-1β Cases (pg/mL)** | **Fatty filum controls (pg/mL)** | **pTB controls (pg/mL)** | **Cases vs fatty filum controls** | **Cases vs pTB controls** |
| --- | --- | --- | --- | --- | --- | --- | --- | --- | --- | --- | --- |
| **Sample Type** | | **Lumbar** | **Vent** | **Lumbar** | **p-value** | **p-value** | **Serum** | **Serum** | **Serum** | **p-value** | **p-value** |
| **Admission** | | n=36 | n=23 | n=11 | p<0.01* | p=0.02* | n=36 | n=11 | n=9 | p=0.2 | p=0.31 |
|  | Median | 6.7 | 0.01 | 0.01 |  |  | 0.01 | 0.01 | 0.01 |  |  |
|  | Min | 0.01 | 0.01 | 0.01 |  |  | 0.01 | 0.01 | 0.01 |  |  |
|  | Max | 58.76 | 27.14 | 0.01 |  |  | 16.37 | 6.89 | 9.95 |  |  |
|  | p25 | 0.01 | 0.01 | 0.01 |  |  | 0.01 | 0.01 | 0.01 |  |  |
|  | p75 | 15.04 | 6.89 | 0.01 |  |  | 3.31 | 0.01 | 0.01 |  |  |
|  | p95 |  |  | 0.01 |  |  |  | 6.89 | 9.95 |  |  |
| **Week 1** | | n=29 | n=9 |  |  |  | n=26 |  |  |  |  |
|  | Median | 0.01 | 0.01 |  |  |  | 0.01 |  |  |  |  |
|  | Min | 0.01 | 0.01 |  |  |  | 0.01 |  |  |  |  |
|  | Max | 99.39 | 50.68 |  |  |  | 13.62 |  |  |  |  |
|  | p25 | 0.01 | 0.01 |  |  |  | 0.01 |  |  |  |  |
|  | p75 | 3.7 | 8.86 |  |  |  | 1.01 |  |  |  |  |
| **Week 2** | | n=30 | n=6 |  |  |  | n=23 |  |  |  |  |
|  | Median | 0.01 | 0.01 |  |  |  | 0.01 |  |  |  |  |
|  | Min | 0.01 | 0.01 |  |  |  | 0.01 |  |  |  |  |
|  | Max | 12.31 | 18 |  |  |  | 14.6 |  |  |  |  |
|  | p25 | 0.01 | 0.01 |  |  |  | 0.01 |  |  |  |  |
|  | p75 | 4.1 | 13.79 |  |  |  | 1.52 |  |  |  |  |
| **Week 3** | | n=21 | n=5 |  |  |  | n=14 |  |  |  |  |
|  | Median | 0.01 | 0.01 |  |  |  | 0.01 |  |  |  |  |
|  | Min | 0.01 | 0.01 |  |  |  | 0.01 |  |  |  |  |
|  | Max | 8.47 | 5.58 |  |  |  | 50.42 |  |  |  |  |
|  | p25 | 0.01 | 0.01 |  |  |  | 0.01 |  |  |  |  |
|  | p75 | 0.01 | 3.8 |  |  |  | 0.01 |  |  |  |  |
| **Week 4** | | n=11 |  |  |  |  | n=7 |  |  |  |  |
|  | Median | 0.01 |  |  |  |  | 0.01 |  |  |  |  |
|  | Min | 0.01 |  |  |  |  | 0.01 |  |  |  |  |
|  | Max | 3.7 |  |  |  |  | 33.5 |  |  |  |  |
|  | p25 | 0.01 |  |  |  |  | 0.01 |  |  |  |  |
|  | p75 | 3.41 |  |  |  |  | 0.01 |  |  |  |  |

Lumbar = lumbar CSF, Vent = ventricular CSF, p= percentile, min = minimum, max = maximum, pTB = pulmonary TB controls, * = statistically significant at *p<0.05,* lumbar and serum samples in week 2 have 1 less sample relative to neuromarkers as this pair of samples was excluded due to a technical error during Luminex analysis, only 2 patients had ventricular CSF samples taken in week 4, due the low sample size biomarker concentrations are not presented

| **Marker** | | **IL-Ra Cases (pg/mL)** | | **IL-Ra controls (pg/mL)** | **Cases vs controls**  **Lumbar** | **Cases vs controls**  **Vent** | **IL-Ra Cases (pg/mL)** | **Fatty filum controls (pg/mL)** | **pTB controls (pg/mL)** | **Cases vs fatty filum controls** | **Cases vs pTB controls** |
| --- | --- | --- | --- | --- | --- | --- | --- | --- | --- | --- | --- |
| **Sample Type** | | **Lumbar** | **Vent** | **Lumbar** | **p-value** | **p-value** | **Serum** | **Serum** | **Serum** | **p-value** | **p-value** |
| **Admission** | | n=36 | n=23 | n=11 | p<0.01* | p<0.01* | n=36 | n=11 | n=9 | p=0.11 | p=0.3 |
|  | Median | 456.2 | 72.65 | 0.01 |  |  | 33.31 | 16.53 | 69.36 |  |  |
|  | Min | 19.02 | 0.01 | 0.01 |  |  | 0.01 | 0.01 | 0.01 |  |  |
|  | Max | 1572.11 | 699.77 | 0.01 |  |  | 223.29 | 80.09 | 279.49 |  |  |
|  | p25 | 204.17 | 28.42 | 0.01 |  |  | 0.01 | 0.01 | 23.65 |  |  |
|  | p75 | 810.35 | 195.84 | 0.01 |  |  | 84.48 | 30.74 | 72.85 |  |  |
|  | p95 |  |  | 0.01 |  |  |  | 80.09 | 279.49 |  |  |
| **Week 1** | | n=29 | n=9 |  |  |  | n=26 |  |  |  |  |
|  | Median | 182.06 | 62.91 |  |  |  | 31.18 |  |  |  |  |
|  | Min | 0.01 | 0.01 |  |  |  | 0.01 |  |  |  |  |
|  | Max | 2074.47 | 252.14 |  |  |  | 619.99 |  |  |  |  |
|  | p25 | 47.26 | 0.01 |  |  |  | 0.01 |  |  |  |  |
|  | p75 | 736.48 | 88.53 |  |  |  | 68.73 |  |  |  |  |
| **Week 2** | | n=30 | n=6 |  |  |  | n=23 |  |  |  |  |
|  | Median | 71.99 | 46.68 |  |  |  | 19.02 |  |  |  |  |
|  | Min | 0.01 | 0.01 |  |  |  | 0.01 |  |  |  |  |
|  | Max | 2060.31 | 154.38 |  |  |  | 184.79 |  |  |  |  |
|  | p25 | 2.53 | 0.01 |  |  |  | 0.01 |  |  |  |  |
|  | p75 | 210.34 | 89.94 |  |  |  | 69.48 |  |  |  |  |
| **Week 3** | | n=21 | n=5 |  |  |  | n=14 |  |  |  |  |
|  | Median | 2.53 | 31.57 |  |  |  | 38.74 |  |  |  |  |
|  | Min | 0.01 | 0.01 |  |  |  | 0.01 |  |  |  |  |
|  | Max | 293.77 | 1492.95 |  |  |  | 384.11 |  |  |  |  |
|  | p25 | 0.01 | 0.01 |  |  |  | 0.01 |  |  |  |  |
|  | p75 | 59.28 | 51.87 |  |  |  | 52.69 |  |  |  |  |
| **Week 4** | | n=11 |  |  |  |  | n=7 |  |  |  |  |
|  | Median | 0.01 |  |  |  |  | 37.8 |  |  |  |  |
|  | Min | 0.01 |  |  |  |  | 0.01 |  |  |  |  |
|  | Max | 618.6 |  |  |  |  | 261.35 |  |  |  |  |
|  | p25 | 0.01 |  |  |  |  | 0.01 |  |  |  |  |
|  | p75 | 130.97 |  |  |  |  | 66.3 |  |  |  |  |

Lumbar = lumbar CSF, Vent = ventricular CSF, p= percentile, min = minimum, max = maximum, pTB = pulmonary TB controls, * = statistically significant at *p<0.05,* lumbar and serum samples in week 2 have 1 less sample relative to neuromarkers as this pair of samples was excluded due to a technical error during Luminex analysis, only 2 patients had ventricular CSF samples taken in week 4, due the low sample size biomarker concentrations are not presented

| **Marker** | | **IL-8 Cases (pg/mL)** | | **IL-8 controls (pg/mL)** | **Cases vs controls**  **Lumbar** | **Cases vs controls**  **Vent** | **IL-8 Cases (pg/mL)** | **Fatty filum controls (pg/mL)** | **pTB controls (pg/mL)** | **Cases vs fatty filum controls** | **Cases vs pTB controls** |
| --- | --- | --- | --- | --- | --- | --- | --- | --- | --- | --- | --- |
| **Sample Type** | | **Lumbar** | **Vent** | **Lumbar** | **p-value** | **p-value** | **Serum** | **Serum** | **Serum** | **p-value** | **p-value** |
| **Admission** | | n=36 | n=23 | n=11 | p<0.01* | p<0.01* | n=36 | n=11 | n=9 | p=0.01* | p=0.55 |
|  | Median | 1981.46 | 490.57 | 6.58 |  |  | 20.14 | 9.93 | 15.37 |  |  |
|  | Min | 487.48 | 23.41 | 3.26 |  |  | 4.31 | 3.47 | 7.41 |  |  |
|  | Max | 6539.84 | 1370.34 | 30.11 |  |  | 280.93 | 42.59 | 300.62 |  |  |
|  | p25 | 1122.93 | 177.8 | 5.07 |  |  | 16.68 | 6.28 | 12.52 |  |  |
|  | p75 | 3396.45 | 806.4 | 13.65 |  |  | 36.2 | 18.75 | 30.44 |  |  |
|  | p95 |  |  | 30.11 |  |  |  | 42.59 | 300.62 |  |  |
| **Week 1** | | n=29 | n=9 |  |  |  | n=26 |  |  |  |  |
|  | Median | 1115.03 | 706.78 |  |  |  | 26.27 |  |  |  |  |
|  | Min | 197.78 | 35.16 |  |  |  | 5.73 |  |  |  |  |
|  | Max | 7766.9 | 3399.34 |  |  |  | 236.15 |  |  |  |  |
|  | p25 | 467.65 | 94.41 |  |  |  | 17.43 |  |  |  |  |
|  | p75 | 2307.95 | 906.85 |  |  |  | 40.62 |  |  |  |  |
| **Week 2** | | n=30 | n=6 |  |  |  | n=23 |  |  |  |  |
|  | Median | 804.1 | 323.85 |  |  |  | 21.77 |  |  |  |  |
|  | Min | 126.07 | 48.82 |  |  |  | 0.01 |  |  |  |  |
|  | Max | 2424.95 | 749.45 |  |  |  | 969.47 |  |  |  |  |
|  | p25 | 407.35 | 182.55 |  |  |  | 13.82 |  |  |  |  |
|  | p75 | 1291.6 | 608.44 |  |  |  | 40.12 |  |  |  |  |
| **Week 3** | | n=21 | n=5 |  |  |  | n=14 |  |  |  |  |
|  | Median | 651.52 | 244.96 |  |  |  | 17.42 |  |  |  |  |
|  | Min | 37.35 | 57.55 |  |  |  | 6.72 |  |  |  |  |
|  | Max | 3329.57 | 805.35 |  |  |  | 108.11 |  |  |  |  |
|  | p25 | 301.26 | 187.91 |  |  |  | 13.91 |  |  |  |  |
|  | p75 | 1355.66 | 354.89 |  |  |  | 28.05 |  |  |  |  |
| **Week 4** | | n=11 |  |  |  |  | n=7 |  |  |  |  |
|  | Median | 635.77 |  |  |  |  | 25.91 |  |  |  |  |
|  | Min | 133.58 |  |  |  |  | 10.84 |  |  |  |  |
|  | Max | 4602.41 |  |  |  |  | 69.42 |  |  |  |  |
|  | p25 | 253.15 |  |  |  |  | 22.19 |  |  |  |  |
|  | p75 | 1099.52 |  |  |  |  | 39.96 |  |  |  |  |

Lumbar = lumbar CSF, Vent = ventricular CSF, p= percentile, min = minimum, max = maximum, pTB = pulmonary TB controls, * = statistically significant at *p<0.05,* lumbar and serum samples in week 2 have 1 less sample relative to neuromarkers as this pair of samples was excluded due to a technical error during Luminex analysis, only 2 patients had ventricular CSF samples taken in week 4, due the low sample size biomarker concentrations are not presented

| **Marker** | | **MCP-1 Cases (pg/mL)** | | **MCP-1 controls (pg/mL)** | **Cases vs controls**  **Lumbar** | **Cases vs controls**  **Vent** | **MCP-1 Cases (pg/mL)** | **Fatty filum controls (pg/mL)** | **pTB controls (pg/mL)** | **Cases vs fatty filum controls** | **Cases vs pTB controls** |
| --- | --- | --- | --- | --- | --- | --- | --- | --- | --- | --- | --- |
| **Sample Type** | | **Lumbar** | **Vent** | **Lumbar** | **p-value** | **p-value** | **Serum** | **Serum** | **Serum** | **p-value** | **p-value** |
| **Admission** | | n=36 | n=23 | n=11 | p<0.01* | p<0.01* | n=36 | n=11 | n=9 | p<0.01* | p<0.01* |
|  | Median | 1947.4 | 4597.47 | 651.38 |  |  | 143.21 | 246.54 | 418.17 |  |  |
|  | Min | 507.8 | 1627.85 | 412.88 |  |  | 20.22 | 198.02 | 160.26 |  |  |
|  | Max | 6257.75 | 15327.69 | 1475.21 |  |  | 684.11 | 610.11 | 1543.51 |  |  |
|  | p25 | 1510.62 | 2678.13 | 533.75 |  |  | 78.21 | 216.59 | 339.71 |  |  |
|  | p75 | 2954.85 | 9227.79 | 1175.25 |  |  | 225.95 | 365.65 | 1084.61 |  |  |
|  | p95 |  |  | 1475.21 |  |  |  | 610.11 | 1543.51 |  |  |
| **Week 1** | | n=29 | n=9 |  |  |  | n=26 |  |  |  |  |
|  | Median | 1280.36 | 2581.72 |  |  |  | 207.33 |  |  |  |  |
|  | Min | 421.86 | 500.45 |  |  |  | 34.4 |  |  |  |  |
|  | Max | 6799.66 | 9936.64 |  |  |  | 792.72 |  |  |  |  |
|  | p25 | 906.29 | 2238.52 |  |  |  | 164.24 |  |  |  |  |
|  | p75 | 1702.15 | 3655.25 |  |  |  | 348.78 |  |  |  |  |
| **Week 2** | | n=30 | n=6 |  |  |  | n=23 |  |  |  |  |
|  | Median | 1466.31 | 4045.46 |  |  |  | 225.1 |  |  |  |  |
|  | Min | 467.46 | 1894.41 |  |  |  | 45.62 |  |  |  |  |
|  | Max | 9046.45 | 11696.31 |  |  |  | 740.67 |  |  |  |  |
|  | p25 | 892.7 | 2395.89 |  |  |  | 143.34 |  |  |  |  |
|  | p75 | 2295.7 | 6962.93 |  |  |  | 326.09 |  |  |  |  |
| **Week 3** | | n=21 | n=5 |  |  |  | n=14 |  |  |  |  |
|  | Median | 1866.22 | 1821.05 |  |  |  | 263.32 |  |  |  |  |
|  | Min | 245.94 | 336.85 |  |  |  | 71.19 |  |  |  |  |
|  | Max | 5474.77 | 4258.12 |  |  |  | 878.79 |  |  |  |  |
|  | p25 | 1006.69 | 1796.2 |  |  |  | 159.45 |  |  |  |  |
|  | p75 | 2455.82 | 2537.43 |  |  |  | 791.14 |  |  |  |  |
| **Week 4** | | n=11 |  |  |  |  | n=7 |  |  |  |  |
|  | Median | 1506.78 |  |  |  |  | 331.02 |  |  |  |  |
|  | Min | 648.07 |  |  |  |  | 95.63 |  |  |  |  |
|  | Max | 2938.7 |  |  |  |  | 866.52 |  |  |  |  |
|  | p25 | 1016.79 |  |  |  |  | 232.79 |  |  |  |  |
|  | p75 | 2539.85 |  |  |  |  | 428.2 |  |  |  |  |

Lumbar = lumbar CSF, Vent = ventricular CSF, p= percentile, min = minimum, max = maximum, pTB = pulmonary TB controls, * = statistically significant at *p<0.05,* lumbar and serum samples in week 2 have 1 less sample relative to neuromarkers as this pair of samples was excluded due to a technical error during Luminex analysis, only 2 patients had ventricular CSF samples taken in week 4, due the low sample size biomarker concentrations are not presented

| **Marker** | | **IP-10 Cases (pg/mL)** | | **IP-10 controls (pg/mL)** | **Cases vs controls**  **Lumbar** | **Cases vs controls**  **Vent** | **IP-10 Cases (pg/mL)** | **Fatty filum controls (pg/mL)** | **pTB controls (pg/mL)** | **Cases vs fatty filum controls** | **Cases vs pTB controls** |
| --- | --- | --- | --- | --- | --- | --- | --- | --- | --- | --- | --- |
| **Sample Type** | | **Lumbar** | **Vent** | **Lumbar** | **p-value** | **p-value** | **Serum** | **Serum** | **Serum** | **p-value** | **p-value** |
| **Admission** | | n=36 | n=23 | n=11 | p<0.01* | p<0.01* | n=36 | n=11 | n=9 | p=0.01* | p<0.01* |
|  | Median | 35505.4 | 24924.98 | 43.42 |  |  | 550.97 | 272.16 | 1931.02 |  |  |
|  | Min | 5919.14 | 970.83 | 7.76 |  |  | 165.43 | 91.43 | 807.44 |  |  |
|  | Max | 77800.54 | 77800.54 | 364.18 |  |  | 3654.24 | 1012.02 | 19318.78 |  |  |
|  | p25 | 25618.52 | 7422.5 | 14.44 |  |  | 395.26 | 186.21 | 996.4 |  |  |
|  | p75 | 69388.13 | 74447.62 | 106.29 |  |  | 1080.1 | 470.15 | 2188.88 |  |  |
|  | p95 |  |  | 364.18 |  |  |  | 1012.02 | 19318.78 |  |  |
| **Week 1** | | n=29 | n=9 |  |  |  | n=26 |  |  |  |  |
|  | Median | 25618.52 | 15672.62 |  |  |  | 463.92 |  |  |  |  |
|  | Min | 1959.68 | 488.21 |  |  |  | 146.81 |  |  |  |  |
|  | Max | 78500.87 | 74531.67 |  |  |  | 4861.21 |  |  |  |  |
|  | p25 | 8734.63 | 1217.97 |  |  |  | 226.1 |  |  |  |  |
|  | p75 | 58255.83 | 69676.58 |  |  |  | 704.59 |  |  |  |  |
| **Week 2** | | n=30 | n=6 |  |  |  | n=23 |  |  |  |  |
|  | Median | 25357.92 | 12102.38 |  |  |  | 324 |  |  |  |  |
|  | Min | 1847.29 | 2804.47 |  |  |  | 162.49 |  |  |  |  |
|  | Max | 76541.2 | 74447.62 |  |  |  | 1582.56 |  |  |  |  |
|  | p25 | 8481.32 | 4147.57 |  |  |  | 194.98 |  |  |  |  |
|  | p75 | 63333.72 | 35471.74 |  |  |  | 507.72 |  |  |  |  |
| **Week 3** | | n=21 | n=5 |  |  |  | n=14 |  |  |  |  |
|  | Median | 14252.21 | 27490.36 |  |  |  | 312.1 |  |  |  |  |
|  | Min | 445.64 | 396.94 |  |  |  | 113.99 |  |  |  |  |
|  | Max | 74447.62 | 74447.62 |  |  |  | 3768.51 |  |  |  |  |
|  | p25 | 3284.14 | 20746.36 |  |  |  | 205.44 |  |  |  |  |
|  | p75 | 25618.52 | 69382.67 |  |  |  | 969.06 |  |  |  |  |
| **Week 4** | | n=11 |  |  |  |  | n=7 |  |  |  |  |
|  | Median | 14017.15 |  |  |  |  | 532.95 |  |  |  |  |
|  | Min | 980.38 |  |  |  |  | 84.56 |  |  |  |  |
|  | Max | 74447.62 |  |  |  |  | 1002.57 |  |  |  |  |
|  | p25 | 2476.6 |  |  |  |  | 358.18 |  |  |  |  |
|  | p75 | 51893.2 |  |  |  |  | 782.34 |  |  |  |  |

Lumbar = lumbar CSF, Vent = ventricular CSF, p= percentile, min = minimum, max = maximum, pTB = pulmonary TB controls, * = statistically significant at *p<0.05,* lumbar and serum samples in week 2 have 1 less sample relative to neuromarkers as this pair of samples was excluded due to a technical error during Luminex analysis, only 2 patients had ventricular CSF samples taken in week 4, due the low sample size biomarker concentrations are not presented

| **Marker** | | **MIP-1α Cases (pg/mL)** | | **MIP-1α controls (pg/mL)** | **Cases vs controls**  **Lumbar** | **Cases vs controls**  **Vent** | **MIP-1α Cases (pg/mL)** | **Fatty filum controls (pg/mL)** | **pTB controls (pg/mL)** | **Cases vs fatty filum controls** | **Cases vs pTB controls** |
| --- | --- | --- | --- | --- | --- | --- | --- | --- | --- | --- | --- |
| **Sample Type** | | **Lumbar** | **Vent** | **Lumbar** | **p-value** | **p-value** | **Serum** | **Serum** | **Serum** | **p-value** | **p-value** |
| **Admission** | | n=36 | n=23 | n=11 | p<0.01* | p<0.01* | n=36 | n=11 | n=9 | p=0.77 | p=0.76 |
|  | Median | 57.79 | 35.2 | 0.01 |  |  | 3.26 | 6.63 | 0.01 |  |  |
|  | Min | 26.48 | 9.54 | 0.01 |  |  | 0.01 | 0.01 | 0.01 |  |  |
|  | Max | 256.37 | 113.17 | 10.61 |  |  | 1694.16 | 63.47 | 78.87 |  |  |
|  | p25 | 41.39 | 24.38 | 0.01 |  |  | 0.01 | 0.01 | 0.01 |  |  |
|  | p75 | 78.92 | 46.58 | 7.41 |  |  | 17.34 | 13.14 | 15.49 |  |  |
|  | p95 |  |  | 10.61 |  |  |  | 63.47 | 78.87 |  |  |
| **Week 1** | | n=29 | n=9 |  |  |  | n=26 |  |  |  |  |
|  | Median | 48.46 | 31.27 |  |  |  | 2.49 |  |  |  |  |
|  | Min | 0.01 | 10.08 |  |  |  | 0.01 |  |  |  |  |
|  | Max | 91.08 | 140.46 |  |  |  | 1516.58 |  |  |  |  |
|  | p25 | 21.5 | 24.62 |  |  |  | 0.01 |  |  |  |  |
|  | p75 | 62.71 | 64.35 |  |  |  | 19.59 |  |  |  |  |
| **Week 2** | | n=30 | n=6 |  |  |  | n=23 |  |  |  |  |
|  | Median | 37.4 | 37.94 |  |  |  | 9.54 |  |  |  |  |
|  | Min | 0.01 | 14.01 |  |  |  | 0.01 |  |  |  |  |
|  | Max | 92 | 49.28 |  |  |  | 864.09 |  |  |  |  |
|  | p25 | 23.3 | 30.09 |  |  |  | 0.01 |  |  |  |  |
|  | p75 | 53.81 | 45.13 |  |  |  | 20.3 |  |  |  |  |
| **Week 3** | | n=21 | n=5 |  |  |  | n=14 |  |  |  |  |
|  | Median | 32.61 | 22.18 |  |  |  | 2.44 |  |  |  |  |
|  | Min | 11.87 | 15.49 |  |  |  | 0.01 |  |  |  |  |
|  | Max | 68.5 | 66.05 |  |  |  | 82.85 |  |  |  |  |
|  | p25 | 22.59 | 21.07 |  |  |  | 0.01 |  |  |  |  |
|  | p75 | 36.28 | 24.27 |  |  |  | 19 |  |  |  |  |
| **Week 4** | | n=11 |  |  |  |  | n=7 |  |  |  |  |
|  | Median | 23.06 |  |  |  |  | 8.81 |  |  |  |  |
|  | Min | 8.49 |  |  |  |  | 0.01 |  |  |  |  |
|  | Max | 65.32 |  |  |  |  | 86.67 |  |  |  |  |
|  | p25 | 17.5 |  |  |  |  | 0.01 |  |  |  |  |
|  | p75 | 46.58 |  |  |  |  | 30.89 |  |  |  |  |

Lumbar = lumbar CSF, Vent = ventricular CSF, p= percentile, min = minimum, max = maximum, pTB = pulmonary TB controls, * = statistically significant at *p<0.05,* lumbar and serum samples in week 2 have 1 less sample relative to neuromarkers as this pair of samples was excluded due to a technical error during Luminex analysis, only 2 patients had ventricular CSF samples taken in week 4, due the low sample size biomarker concentrations are not presented

| **Marker** | | **GRO Cases (pg/mL)** | | **GRO controls (pg/mL)** | **Cases vs controls**  **Lumbar** | **Cases vs controls**  **Vent** | **GRO Cases** | **Fatty filum controls (pg/mL)** | **pTB controls (pg/mL)** | **Cases vs fatty filum controls** | **Cases vs pTB controls** |
| --- | --- | --- | --- | --- | --- | --- | --- | --- | --- | --- | --- |
| **Sample Type** | | **Lumbar** | **Vent** | **Lumbar** | **p-value** | **p-value** | **Serum** | **Serum** | **Serum** | **p-value** | **p-value** |
| **Admission** | | n=36 | n=23 | n=11 | p<0.01* | p<0.01* | n=36 | n=11 | n=9 | p=0.28 | p=0.67 |
|  | Median | 1431.18 | 166.77 | 0.01 |  |  | 2165.63 | 2273.49 | 2414.41 |  |  |
|  | Min | 250.17 | 0.01 | 0.01 |  |  | 873 | 328.69 | 1254.55 |  |  |
|  | Max | 3207.74 | 819.52 | 0.01 |  |  | 4696.52 | 3272.34 | 3062.44 |  |  |
|  | p25 | 1010.29 | 43.9 | 0.01 |  |  | 1793 | 1149.74 | 1538.76 |  |  |
|  | p75 | 1852.98 | 358.33 | 0.01 |  |  | 2779.23 | 2560.79 | 2816.48 |  |  |
|  | p95 |  |  | 0.01 |  |  |  | 3272.34 | 3062.44 |  |  |
| **Week 1** | | n=29 | n=9 |  |  |  | n=26 |  |  |  |  |
|  | Median | 819.18 | 109.88 |  |  |  | 2155.46 |  |  |  |  |
|  | Min | 130.51 | 0.01 |  |  |  | 127.94 |  |  |  |  |
|  | Max | 4067.42 | 3269.84 |  |  |  | 3651.44 |  |  |  |  |
|  | p25 | 429.08 | 0.01 |  |  |  | 1274.39 |  |  |  |  |
|  | p75 | 1589.94 | 415.05 |  |  |  | 2859.11 |  |  |  |  |
| **Week 2** | | n=30 | n=6 |  |  |  | n=23 |  |  |  |  |
|  | Median | 696.18 | 179.1 |  |  |  | 2385.49 |  |  |  |  |
|  | Min | 62.24 | 0.01 |  |  |  | 1030.95 |  |  |  |  |
|  | Max | 2194.12 | 502.87 |  |  |  | 4252.57 |  |  |  |  |
|  | p25 | 320.24 | 38.72 |  |  |  | 1909.51 |  |  |  |  |
|  | p75 | 1267.34 | 290.01 |  |  |  | 3237.48 |  |  |  |  |
| **Week 3** | | n=21 | n=5 |  |  |  | n=14 |  |  |  |  |
|  | Median | 508.08 | 107.09 |  |  |  | 2530.25 |  |  |  |  |
|  | Min | 0.01 | 0.01 |  |  |  | 956.73 |  |  |  |  |
|  | Max | 2898.23 | 180.76 |  |  |  | 5093.25 |  |  |  |  |
|  | p25 | 348.11 | 73.41 |  |  |  | 1834.52 |  |  |  |  |
|  | p75 | 1351.99 | 160.21 |  |  |  | 2984.26 |  |  |  |  |
| **Week 4** | | n=11 |  |  |  |  | n=7 |  |  |  |  |
|  | Median | 931.36 |  |  |  |  | 2609.84 |  |  |  |  |
|  | Min | 77.43 |  |  |  |  | 1334.27 |  |  |  |  |
|  | Max | 2425.63 |  |  |  |  | 3242.75 |  |  |  |  |
|  | p25 | 277.25 |  |  |  |  | 1597.04 |  |  |  |  |
|  | p75 | 1785.63 |  |  |  |  | 2948.99 |  |  |  |  |

Lumbar = lumbar CSF, Vent = ventricular CSF, p= percentile, min = minimum, max = maximum, pTB = pulmonary TB controls, * = statistically significant at *p<0.05,* lumbar and serum samples in week 2 have 1 less sample relative to neuromarkers as this pair of samples was excluded due to a technical error during Luminex analysis, only 2 patients had ventricular CSF samples taken in week 4, due the low sample size biomarker concentrations are not presented

| **Marker** | | **IL-10 Cases (pg/mL)** | | **IL-10 controls (pg/mL)** | **Cases vs controls**  **Lumbar** | **Cases vs controls**  **Vent** | **IL-10 Cases (pg/mL)** | **Fatty filum controls (pg/mL)** | **pTB controls (pg/mL)** | **Cases vs fatty filum controls** | **Cases vs pTB controls** |
| --- | --- | --- | --- | --- | --- | --- | --- | --- | --- | --- | --- |
| **Sample Type** | | **Lumbar** | **Vent** | **Lumbar** | **p-value** | **p-value** | **Serum** | **Serum** | **Serum** | **p-value** | **p-value** |
| **Admission** | | n=36 | n=23 | n=11 | p<0.01* | p<0.01* | n=36 | n=11 | n=9 | p=0.83 | p=0.07 |
|  | Median | 420.32 | 97.22 | 0.01 |  |  | 8.54 | 8.01 | 19.31 |  |  |
|  | Min | 107.18 | 41.61 | 0.01 |  |  | 0.01 | 0.01 | 4.08 |  |  |
|  | Max | 1209.5 | 536.41 | 0.01 |  |  | 78.76 | 31.98 | 44.67 |  |  |
|  | p25 | 252.45 | 61.66 | 0.01 |  |  | 1.78 | 3.4 | 7.58 |  |  |
|  | p75 | 653.04 | 255.17 | 0.01 |  |  | 17.86 | 18.93 | 32.26 |  |  |
|  | p95 |  |  | 0.01 |  |  |  | 31.98 | 44.67 |  |  |
| **Week 1** | | n=29 | n=9 |  |  |  | n=26 |  |  |  |  |
|  | Median | 140.29 | 50.24 |  |  |  | 14.04 |  |  |  |  |
|  | Min | 46.45 | 11.47 |  |  |  | 0.01 |  |  |  |  |
|  | Max | 873.74 | 603.21 |  |  |  | 85.51 |  |  |  |  |
|  | p25 | 108.71 | 29.09 |  |  |  | 5.47 |  |  |  |  |
|  | p75 | 234.29 | 153.16 |  |  |  | 27.43 |  |  |  |  |
| **Week 2** | | n=30 | n=6 |  |  |  | n=23 |  |  |  |  |
|  | Median | 156.3 | 55.89 |  |  |  | 16.69 |  |  |  |  |
|  | Min | 9.73 | 34.79 |  |  |  | 0.01 |  |  |  |  |
|  | Max | 439.5 | 605.45 |  |  |  | 132.23 |  |  |  |  |
|  | p25 | 84.18 | 35.62 |  |  |  | 4.92 |  |  |  |  |
|  | p75 | 251.34 | 518.97 |  |  |  | 36.53 |  |  |  |  |
| **Week 3** | | n=21 | n=5 |  |  |  | n=14 |  |  |  |  |
|  | Median | 156.41 | 108.99 |  |  |  | 5.87 |  |  |  |  |
|  | Min | 22.22 | 27.07 |  |  |  | 0.01 |  |  |  |  |
|  | Max | 291.39 | 267.55 |  |  |  | 159.74 |  |  |  |  |
|  | p25 | 95.38 | 64.57 |  |  |  | 0.01 |  |  |  |  |
|  | p75 | 215.3 | 154.01 |  |  |  | 25.74 |  |  |  |  |
| **Week 4** | | n=11 |  |  |  |  | n=7 |  |  |  |  |
|  | Median | 216.77 |  |  |  |  | 16.88 |  |  |  |  |
|  | Min | 74.58 |  |  |  |  | 8.41 |  |  |  |  |
|  | Max | 275.68 |  |  |  |  | 68.74 |  |  |  |  |
|  | p25 | 125.43 |  |  |  |  | 12.38 |  |  |  |  |
|  | p75 | 254.48 |  |  |  |  | 38.48 |  |  |  |  |

Lumbar = lumbar CSF, Vent = ventricular CSF, p= percentile, min = minimum, max = maximum, pTB = pulmonary TB controls, * = statistically significant at *p<0.05,* lumbar and serum samples in week 2 have 1 less sample relative to neuromarkers as this pair of samples was excluded due to a technical error during Luminex analysis, only 2 patients had ventricular CSF samples taken in week 4, due the low sample size biomarker concentrations are not presented

| **Marker** | | **VEGF Cases (pg/mL)** | | **VEGF controls (pg/mL)** | **Cases vs controls**  **Lumbar** | **Cases vs controls**  **Vent** | **VEGF Cases (pg/mL)** | **Fatty filum controls (pg/mL)** | **pTB controls (pg/mL)** | **Cases vs fatty filum controls** | **Cases vs pTB controls** |
| --- | --- | --- | --- | --- | --- | --- | --- | --- | --- | --- | --- |
| **Sample Type** | | **Lumbar** | **Vent** | **Lumbar** | **p-value** | **p-value** | **Serum** | **Serum** | **Serum** | **p-value** | **p-value** |
| **Admission** | | n=36 | n=23 | n=11 | p<0.01* | p<0.01* | n=36 | n=11 | n=9 | p=0.59 | p=0.27 |
|  | Median | 145.33 | 136.15 | 0.01 |  |  | 220.27 | 327.99 | 383.57 |  |  |
|  | Min | 0.01 | 0.01 | 0.01 |  |  | 0.01 | 0.01 | 0.01 |  |  |
|  | Max | 836.7 | 424.78 | 0.01 |  |  | 1442.94 | 816.74 | 1950.31 |  |  |
|  | p25 | 108 | 0.01 | 0.01 |  |  | 152.5 | 90.24 | 220.27 |  |  |
|  | p75 | 269.13 | 220.27 | 0.01 |  |  | 382.75 | 546.56 | 681.05 |  |  |
|  | p95 |  |  | 0.01 |  |  |  | 816.74 | 1950.31 |  |  |
| **Week 1** | | n=29 | n=9 |  |  |  | n=26 |  |  |  |  |
|  | Median | 141.88 | 67.17 |  |  |  | 236.19 |  |  |  |  |
|  | Min | 0.01 | 0.01 |  |  |  | 0.01 |  |  |  |  |
|  | Max | 558.81 | 376.55 |  |  |  | 729.24 |  |  |  |  |
|  | p25 | 97.28 | 0.01 |  |  |  | 130.44 |  |  |  |  |
|  | p75 | 194.76 | 166.22 |  |  |  | 358.31 |  |  |  |  |
| **Week 2** | | n=30 | n=6 |  |  |  | n=23 |  |  |  |  |
|  | Median | 139.7 | 142.49 |  |  |  | 247.01 |  |  |  |  |
|  | Min | 0.01 | 0.01 |  |  |  | 0.01 |  |  |  |  |
|  | Max | 504.41 | 399.71 |  |  |  | 973.74 |  |  |  |  |
|  | p25 | 0.01 | 56.6 |  |  |  | 162.47 |  |  |  |  |
|  | p75 | 206.25 | 309 |  |  |  | 383.57 |  |  |  |  |
| **Week 3** | | n=21 | n=5 |  |  |  | n=14 |  |  |  |  |
|  | Median | 0.01^a^ | 238.68 |  |  |  | 180.6 |  |  |  |  |
|  | Min | 0.01 | 97.28 |  |  |  | 0.01 |  |  |  |  |
|  | Max | 558.81 | 327.99 |  |  |  | 819.39 |  |  |  |  |
|  | p25 | 0.01 | 221.98 |  |  |  | 150.43 |  |  |  |  |
|  | p75 | 184.11 | 271.79 |  |  |  | 265.75 |  |  |  |  |
| **Week 4** | | n=11 |  |  |  |  | n=7 |  |  |  |  |
|  | Median | 90.24 |  |  |  |  | 166.22 |  |  |  |  |
|  | Min | 0.01 |  |  |  |  | 0.01 |  |  |  |  |
|  | Max | 399.71 |  |  |  |  | 970.41 |  |  |  |  |
|  | p25 | 0.01 |  |  |  |  | 135.06 |  |  |  |  |
|  | p75 | 127.84 |  |  |  |  | 534.92 |  |  |  |  |

Lumbar = lumbar CSF, Vent = ventricular CSF, p= percentile, min = minimum, max = maximum, pTB = pulmonary TB controls, * = statistically significant at *p<0.05,* ^a^ The LLOD for VEGF was 80, therefore values <80 were assigned 0.01; therefore the median in week 3 appears much lower than the week 2 and week 4 medians, only 2 patients had ventricular CSF samples taken in week 4, due the low sample size biomarker concentrations are not presented

| **Marker** | | **RANTES Cases (pg/mL)** | | **RANTES controls (pg/mL)** | **Cases vs controls**  **Lumbar** | **Cases vs controls**  **Vent** | **RANTES Cases (pg/mL)** | **Fatty filum controls (pg/mL)** | **pTB controls (pg/mL)** | **Cases vs fatty filum controls** | **Cases vs pTB controls** |
| --- | --- | --- | --- | --- | --- | --- | --- | --- | --- | --- | --- |
| **Sample Type** | | **Lumbar** | **Vent** | **Lumbar** | **p-value** | **p-value** | **Serum** | **Serum** | **Serum** | **p-value** | **p-value** |
| **Admission** | | n=36 | n=23 | n=11 | p=0.1 |  | n=36 | n=11 | n=9 | p=0.1 | p<0.01 |
|  | Median | 0.01 | 0.01 | 0.01 |  |  | 4375.69 | 7270.56 | 9843.51 |  |  |
|  | Min | 0.01 | 0.01 | 0.01 |  |  | 1356.4 | 3511.32 | 4984.65 |  |  |
|  | Max | 1022.21 | 0.01 | 0.01 |  |  | 13288.76 | 12311.36 | 12977.91 |  |  |
|  | p25 | 0.01 | 0.01 | 0.01 |  |  | 3680.88 | 4008.91 | 7407.29 |  |  |
|  | p75 | 0.01 | 0.01 | 0.01 |  |  | 7155.71 | 9864.55 | 11179.14 |  |  |
|  | p95 |  |  | 0.01 |  |  |  | 12311.36 | 12977.91 |  |  |
| **Week 1** | | n=29 | n=9 |  |  |  | n=26 |  |  |  |  |
|  | Median | 0.01 | 0.01 |  |  |  | 4970.54 |  |  |  |  |
|  | Min | 0.01 | 0.01 |  |  |  | 0.01 |  |  |  |  |
|  | Max | 412.04 | 0.01 |  |  |  | 12984.16 |  |  |  |  |
|  | p25 | 0.01 | 0.01 |  |  |  | 4164.56 |  |  |  |  |
|  | p75 | 0.01 | 0.01 |  |  |  | 8418.42 |  |  |  |  |
| **Week 2** | | n=30 | n=6 |  |  |  | n=23 |  |  |  |  |
|  | Median | 0.01 | 0.01 |  |  |  | 5026.29 |  |  |  |  |
|  | Min | 0.01 | 0.01 |  |  |  | 862.17 |  |  |  |  |
|  | Max | 314.6 | 0.01 |  |  |  | 11374.73 |  |  |  |  |
|  | p25 | 0.01 | 0.01 |  |  |  | 3800.18 |  |  |  |  |
|  | p75 | 0.01 | 0.01 |  |  |  | 8559.62 |  |  |  |  |
| **Week 3** | | n=21 | n=5 |  |  |  | n=14 |  |  |  |  |
|  | Median | 0.01 | 0.01 |  |  |  | 4851.89 |  |  |  |  |
|  | Min | 0.01 | 0.01 |  |  |  | 1615.88 |  |  |  |  |
|  | Max | 0.01 | 0.01 |  |  |  | 11787.36 |  |  |  |  |
|  | p25 | 0.01 | 0.01 |  |  |  | 4037.31 |  |  |  |  |
|  | p75 | 0.01 | 0.01 |  |  |  | 7747.56 |  |  |  |  |
| **Week 4** | | n=11 |  |  |  |  | n=7 |  |  |  |  |
|  | Median | 0.01 |  |  |  |  | 4403.37 |  |  |  |  |
|  | Min | 0.01 |  |  |  |  | 2381.16 |  |  |  |  |
|  | Max | 0.01 |  |  |  |  | 13632.33 |  |  |  |  |
|  | p25 | 0.01 |  |  |  |  | 3670.28 |  |  |  |  |
|  | p75 | 0.01 |  |  |  |  | 9796.18 |  |  |  |  |

Lumbar = lumbar CSF, Vent = ventricular CSF, p= percentile, min = minimum, max = maximum, pTB = pulmonary TB controls, * = statistically significant at *p<0.05,* lumbar and serum samples in week 2 have 1 less sample relative to neuromarkers as this pair of samples was excluded due to a technical error during Luminex analysis, only 2 patients had ventricular CSF samples taken in week 4, due the low sample size biomarker concentrations are not presented

**
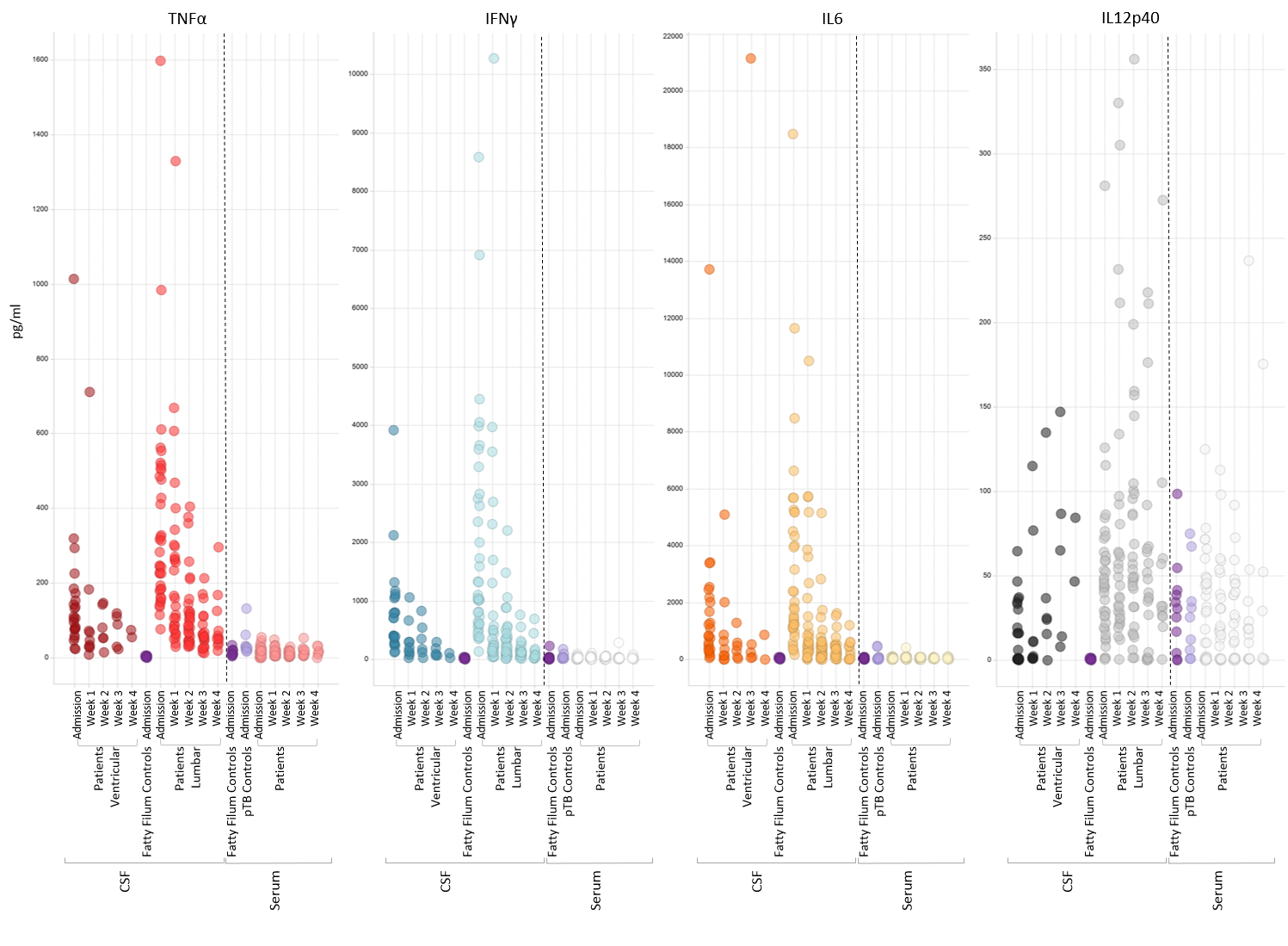
Supplement 5: Inflammatory marker temporal profile plots**


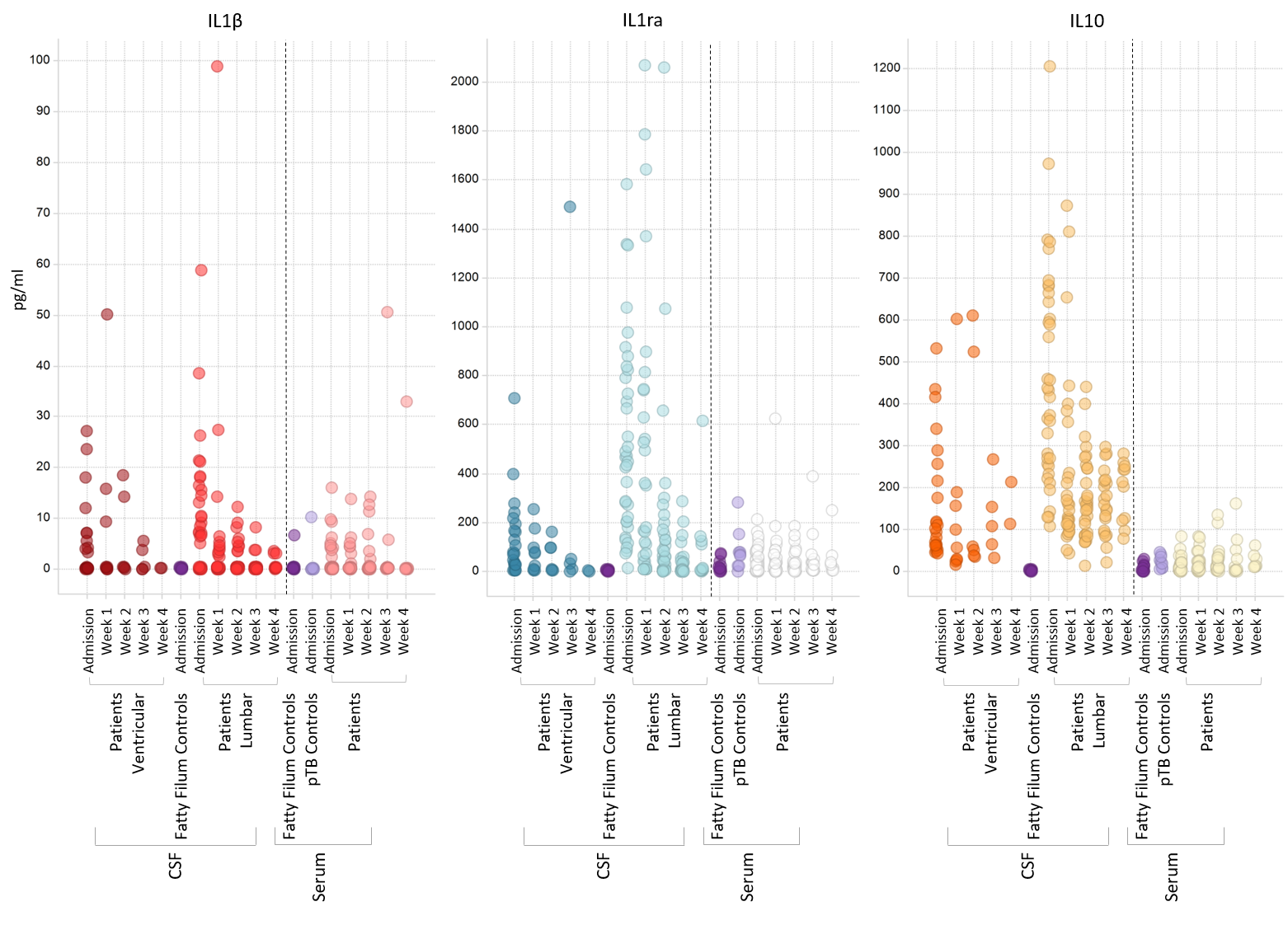


**
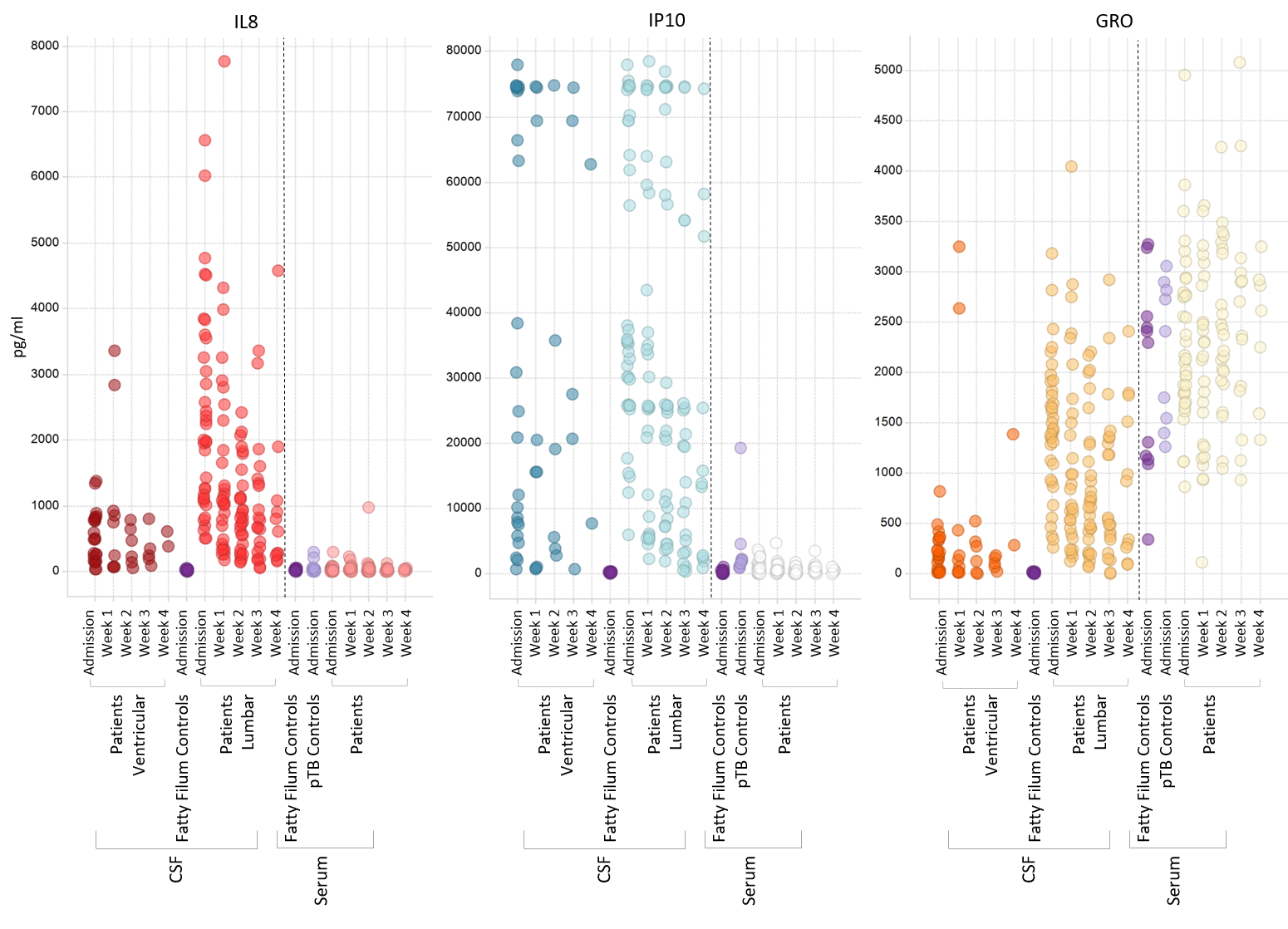

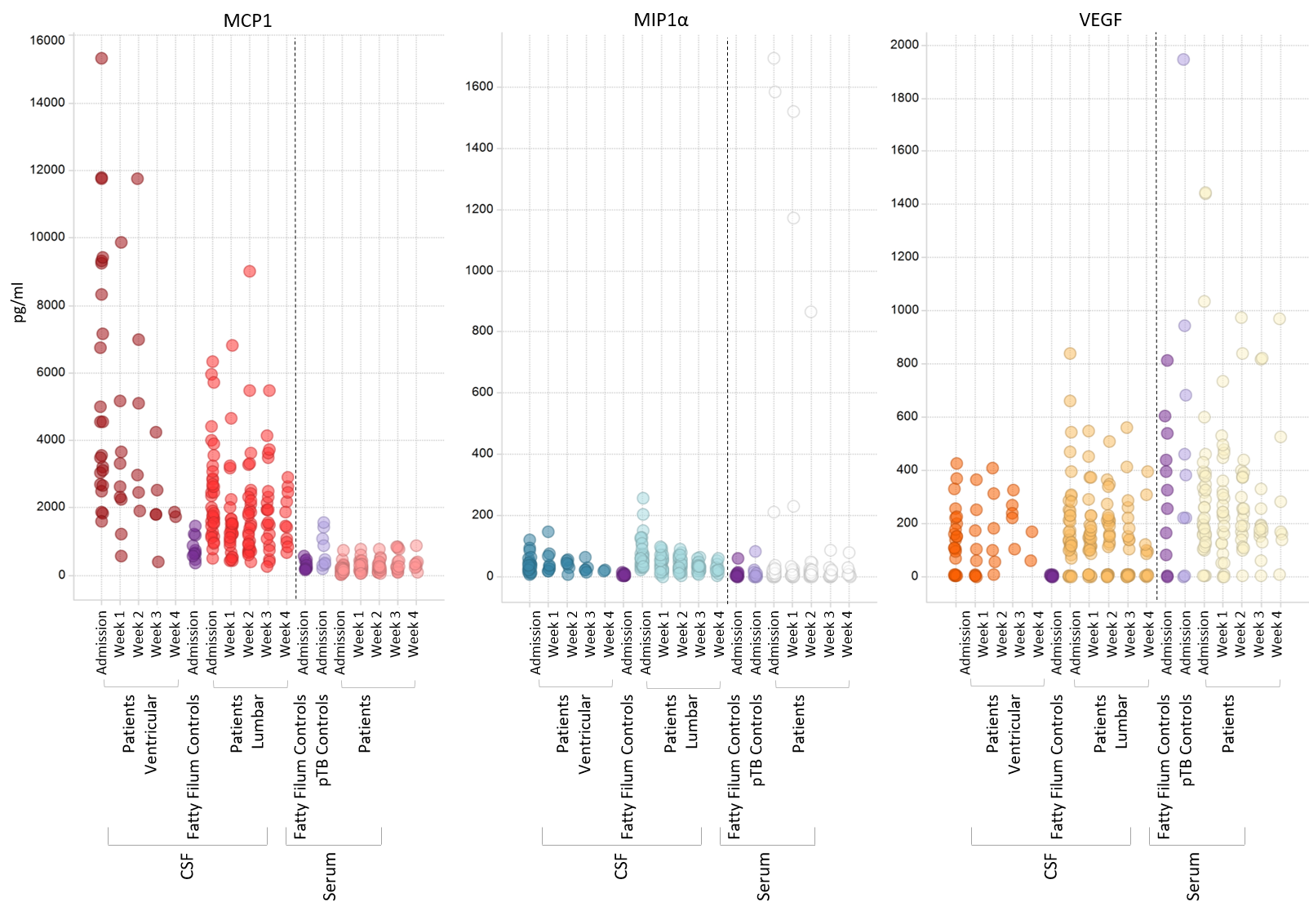
**

**Supplement 6: PCA bi-plots**

Principal component analysis biplots demonstrated neuro- and inflammatory marker concentrations for cases and controls across sample types.

**Neuro-markers concentrations: cases versus controls**

B

A

**
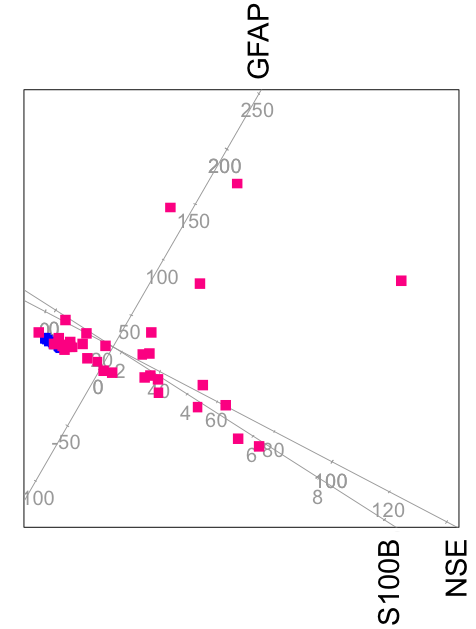
**

S100B

GFAP

NSE

TBM Cases serum

Fatty filum controls serum

pTB controls serum

GFAP

NSE

S100BB

TBM Cases ventricular CSF

Fatty filum controls

C

TBM Cases lumbar CSF

Fatty filum controls

C

These PCA bi-plots demonstrate admission S100B, NSE and GFAP concentrations simultaneously. Markers on the graphs represent individual patients/controls and their concentration (ng/ml) of S100B, NSE and GFAP is indicated by their distance from the 3 axes. **A**: Lumbar CSF concentrations of neuromarkers for TBM cases (pink) and fatty filum controls (blue). Cases had higher concentrations for all neuromarkers. **B**: Ventricular CSF concentrations of neuromarkers for TBM cases (green) and controls (blue). Cases had higher concentrations for all neuromarkers. **C**: Serum concentrations for TBM cases (orange), fatty filum controls (dark blue), and pulmonary TB (pTB) controls (light purple markers). Serum concentrations of all 3 neuromarkers in controls and TBM cases overlapped, indicating that TBM serum neuromarker concentrations were not significantly different from control concentrations.

**Inflammatory marker concentrations: cases versus controls**

A

B


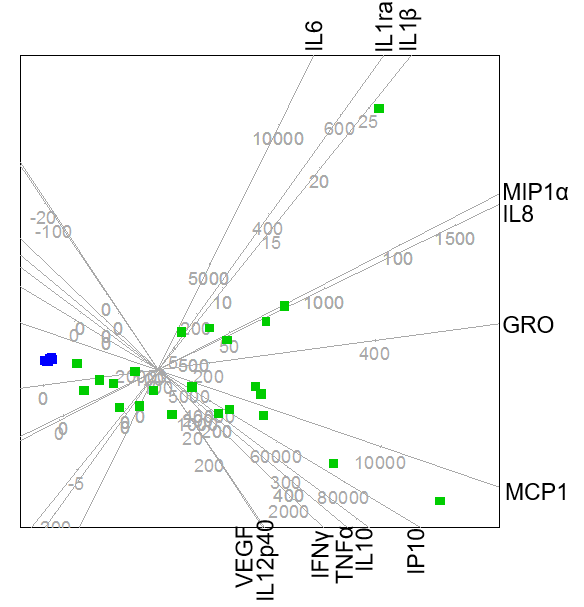

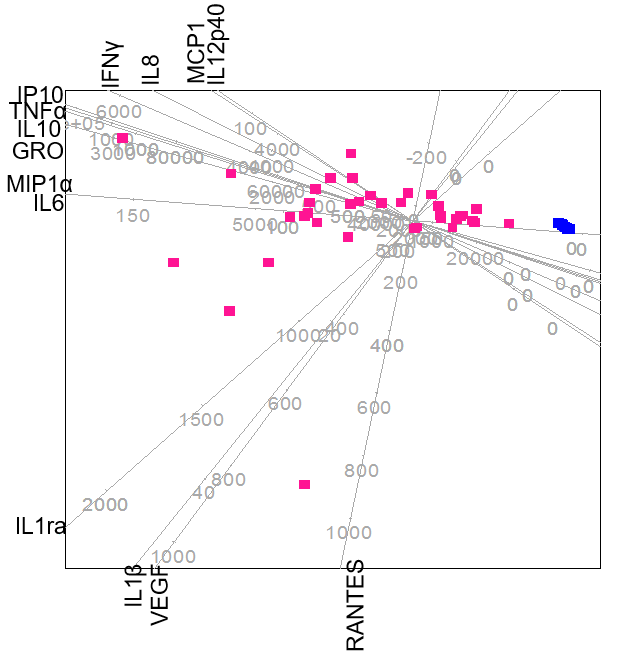


C

These PCA bi-plots demonstrate admission inflammatory marker concentrations simultaneously. Markers on the graphs represent individual patients/controls and their concentration (pg/mL) of inflammatory markers are indicated by the distance from the 3 axes. **A**: Lumbar CSF concentrations of inflammatory markers for TBM cases (pink) and fatty filum controls (blue). Cases had higher concentrations for all inflammatory markers. **B**: Ventricular CSF concentrations for TBM cases (green) and controls (blue). Cases had higher concentrations on all inflammatory markers. **C**: Serum concentrations for TBM cases (orange), fatty filum controls (dark blue), and pulmonary TB (pTB) controls (light purple markers) - control and TBM case concentrations overlapped, indicating that TBM serum inflammatory marker concentrations were not significantly different from control concentrations.

TBM Cases lumbar CSF Fatty filum controls

TBM Cases ventricular CSF Fatty filum controls

IL12p40

IL-1beta

C

TBM Cases serum

Fatty filum controls serum

pTB controls serum

**Biomarker concentrations across the 3 sample types for TBM cases**


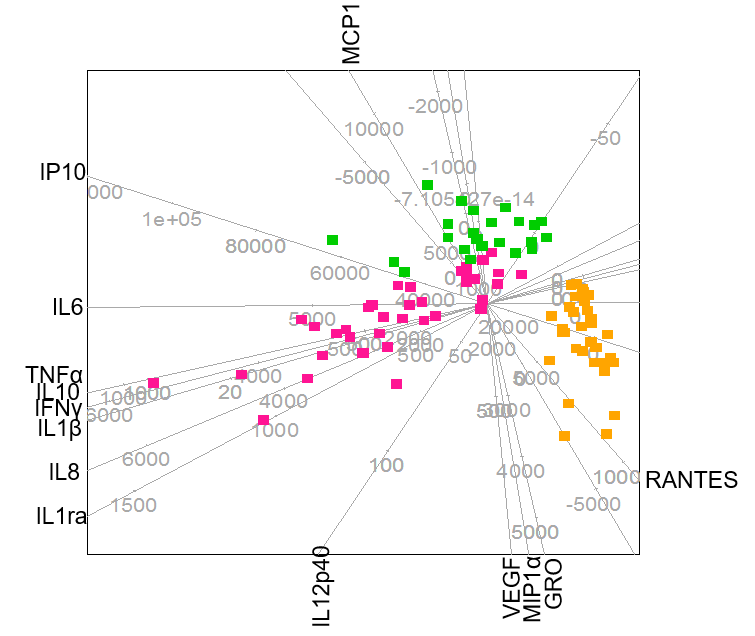

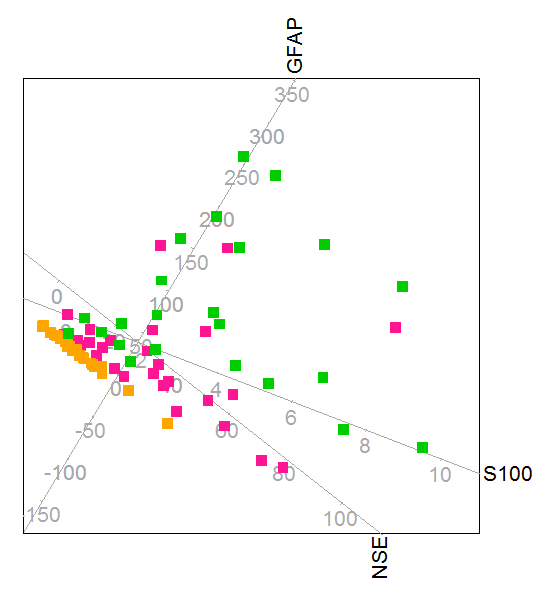


Serum Lumbar CSF Ventricular CSF

A

B

These PCA bi-plots demonstrate admission lumbar CSF (pink), ventricular CSF (green), and serum (orange) biomarker concentrations for TBM cases simultaneously as described for Figures 2-3. **A**: For all 3 neuromarkers ventricular CSF concentrations were greater than lumbar CSF which were greater than serum concentrations. **B**: lumbar and ventricular CSF concentrations exceeded serum concentrations for all 14 inflammatory markers, and lumbar CSF concentrations were greater than ventricular CSF for most inflammatory markers.
